# Functional brain defects in a mouse model of a chromosomal t(1;11) translocation that disrupts *DISC1* and confers increased risk of psychiatric illness

**DOI:** 10.1038/s41398-021-01256-3

**Published:** 2021-02-19

**Authors:** Marion Bonneau, Shane T. O’ Sullivan, Miguel A. Gonzalez-Lozano, Paul Baxter, Phillippe Gautier, Elena Marchisella, Neil R. Hardingham, Robert A. Chesters, Helen Torrance, David M. Howard, Maurits A. Jansen, Melanie McMillan, Yasmin Singh, Michel Didier, Frank Koopmans, Colin A. Semple, Andrew M. McIntosh, Hansjürgen Volkmer, Maarten Loos, Kevin Fox, Giles E. Hardingham, Anthony C. Vernon, David J. Porteous, August B. Smit, David J. Price, J. Kirsty Millar

**Affiliations:** 1 Centre for Genomic and Experimental Medicine, MRC Institute of Genetics and Molecular Medicine at the University of Edinburgh, Edinburgh, UK; 2 Department of Molecular and Cellular Neurobiology, Center for Neurogenomics and Cognitive Research, VU University, Amsterdam, The Netherlands; 3 Centre for Discovery Brain Sciences, Hugh Robson Building, The University of Edinburgh, Edinburgh, UK; 4 UK Dementia Research Institute, Edinburgh Medical School, The University of Edinburgh, Edinburgh, UK; 5 MRC Human Genetics Unit, MRC Institute of Genetics and Molecular Medicine at the University of Edinburgh, Edinburgh, UK; 6 Sylics Synaptologics BV, Amsterdam, The Netherlands; 7 School of Biosciences, Museum Avenue, Cardiff University, Cardiff, UK; 8 Department of Basic and Clinical Neuroscience, Institute of Psychiatry, Psychology and Neuroscience, King’s College London, London, UK; 9 Social, Genetic and Developmental Psychiatry Centre, Institute of Psychiatry, Psychology & Neuroscience, King’s College London, London, UK; 10 Division of Psychiatry, Kennedy Tower, The University of Edinburgh, Edinburgh, UK; 11 Edinburgh Preclinical Imaging, The Chancellor’s Building, The University of Edinburgh, Edinburgh, UK; 12 Centre for Reproductive Health, The Queen’s Medical Research Institute, The University of Edinburgh, Edinburgh, UK; 13 Centre for Genomics and Transcriptomics, Paul-Ehrlich-Straße 23, Tübingen, Germany; 14 Translational Sciences at Sanofi, Chilly-Mazarin, France; 15 Department of Molecular Biology, NMI Natural and Medical Sciences Institute at the University of Tübingen, Reutlingen, Germany; 16 MRC Centre for Neurodevelopmental Disorders, King’s College London, London, UK

**Keywords:** Molecular neuroscience, Psychology

## Abstract

A balanced t(1;11) translocation that directly disrupts DISC1 is linked to schizophrenia and affective disorders. We previously showed that a mutant mouse, named Der1, recapitulates the effect of the translocation upon DISC1 expression. Here, RNAseq analysis of Der1 mouse brain tissue found enrichment for dysregulation of the same genes and molecular pathways as in neuron cultures generated previously from human t(1;11) translocation carriers via the induced pluripotent stem cell route. DISC1 disruption therefore apparently accounts for a substantial proportion of the effects of the t(1;11) translocation. RNAseq and pathway analysis of the mutant mouse predicts multiple Der1-induced alterations converging upon synapse function and plasticity. Synaptosome proteomics confirmed that the Der1 mutation impacts synapse composition, and electrophysiology found reduced AMPA:NMDA ratio in hippocampal neurons, indicating changed excitatory signalling. Moreover, hippocampal parvalbumin-positive interneuron density is increased, suggesting that the Der1 mutation affects inhibitory control of neuronal circuits. These phenotypes predict that neurotransmission is impacted at many levels by DISC1 disruption in human t(1;11) translocation carriers. Notably, genes implicated in schizophrenia, depression and bipolar disorder by large-scale genetic studies are enriched among the Der1-dysregulated genes, just as we previously observed for the t(1;11) translocation carrier-derived neurons. Furthermore, RNAseq analysis predicts that the Der1 mutation primarily targets a subset of cell types, pyramidal neurons and interneurons, previously shown to be vulnerable to the effects of common schizophrenia-associated genetic variants. In conclusion, DISC1 disruption by the t(1;11) translocation may contribute to the psychiatric disorders of translocation carriers through commonly affected pathways and processes in neurotransmission.

## Introduction

Psychiatric illnesses such as schizophrenia and recurrent affective disorders have a substantial underlying genetic component. Considerable progress has been made in recent years towards the identification of the multitude of genes involved using large-scale studies of genome-wide association (GWAS) and recurrent copy number variants (CNVs)^1–6^. GWAS tends to identify genomic loci with common, but small, individual effects that encompass several genes, leaving the specific causal genes unidentified, unless further refinements are applied. In contrast, recurrent CNVs are rare, tending to exert a strong effect (most likely due to large changes in expression levels of the genes at fault), but also usually encompass multiple genes. Chromosomal rearrangements, such as translocations, linked to psychiatric disorders, are rarer still but can have the advantage of strong effects and accurate pinpointing of genes due to their disruption by the breakpoints of the rearranged genomic segments. It is likely that convergence of data arising from genomic events such as these will assist in revealing the genes and mechanisms that predispose to major mental illness.

One example of a chromosomal rearrangement linked to psychiatric disorders is a t(1;11) translocation that substantially increases the risk of developing schizophrenia or affective disorders in a large Scottish family^7–9^. The psychiatric symptoms presented by t(1;11) translocation carriers are typical, that is, they are within the range of current diagnostic criteria, and are accompanied by reduced white matter integrity^10^, cortical thickness^11^ and prefrontal cortex gyrification^9^, all types of schizophrenia. Carriers of the t(1;11) translocation also have decreased glutamate levels in the dorsolateral prefrontal cortex^9^. Moreover, transcriptome analysis of induced pluripotent stem cell (IPSC)-derived cortical neurons from t(1;11) translocation carriers^12^ found enrichment for dysregulated genes at putative schizophrenia and depression loci discovered through large-scale GWAS and CNV studies^1–3^, potentially identifying some of the genes of interest at those loci, and indicating that the t(1;11) translocation may trigger disease pathways shared with schizophrenic patients who are not translocation carriers.

The t(1;11) translocation directly disrupts the *DISC1* gene on chromosome 1^13^. *DISC1* encodes a potential molecular scaffold protein involved in multiple critical functions in the developing and adult brain^14,15^, including neurogenesis^16–18^, neuronal cargo trafficking^19–23^ and neurotransmission^23–26^. *DISC1* disruption is therefore likely to contribute substantially to mechanisms leading to psychiatric illness in t(1;11) translocation carriers.

Two apparently non-coding genes of unknown function, *DISC2* and *DISC1FP1* (otherwise known as *Boymaw*), are also disrupted on chromosomes 1 and 11, respectively^13,27^, and potential genetic modifier loci have been identified within the family^28^, all of which may additionally impact disease mechanisms in t(1;11) translocation carriers. It is now important to discover how each of these disruptions and putative modifiers relates to the gene expression changes, brain structure alterations and psychiatric symptoms of t(1;11) translocation carriers.

To examine the impact of *DISC1* disruption in isolation from the additional complexities of *DISC2* disruption and loss of normal *DISC1FP1* function, and of potential genetic modifiers, we have utilised a mutant mouse that accurately recapitulates the effects of the translocation upon DISC1 expression^12^. IPSC-derived neural precursor cells and cortical neurons from t(1;11) translocation carriers exhibit reduced *DISC1* expression^12^. Chimeric transcripts encoding aberrant C-terminally truncated chimeric forms of DISC1 are also produced in the IPSC-derived neural cells as a result of the fusion between the *DISC1* and *DISC1FP1* genes on the derived chromosome 1^12,29^. The mutant mouse was precisely engineered to mimic the fusion between *DISC1* and *DISC1FP1* on the derived 1 chromosome and exhibits reduced Disc1 levels plus chimeric transcript expression^12^. This mutant mouse is referred to as *Der1*.

Heterozygous *Der1* mice express reduced levels of wild-type Disc1 plus the aberrant chimeric transcripts^12,29^. Because Disc1 multimerises^30^, there is thus potential in heterozygotes for dominant-negative effects due to interaction between wild-type and mutant Disc1. Homozygotes, however, lack any wild-type Disc1 and may express high levels of aberrant Disc1. Despite heterozygotes corresponding most closely to t(1:11) translocation carriers, we opted to study both mutant genotypes in order to obtain the most complete understanding of the likely effects of DISC1 disruption. A flowchart (Supplementary Fig. 1) illustrates the experimental approach taken, with the aim of allowing the results described here to be compared with previously published t(1:11) translocation studies and integrated with psychiatric genetic association studies of single-nucleotide polymorphism (SNP) and CNV variants in the general population. We combine magnetic resonance imaging (MRI), histology, transcriptomics, synaptosome proteomics and electrophysiology to demonstrate that the *Der1* mutation primarily affects cellular properties rather than brain structure and that it targets a variety of cell types including neurons. Patterns of gene expression and predictions of altered biological processes substantially overlap between *Der1* cortex and IPSC-derived cortical neuron cultures from t(1:11) translocation carriers. We find widespread dysregulation of genes implicated as potential common risk factors for schizophrenia, depression and bipolar disorder. We, therefore, propose that *DISC1* disruption targets common pathways shared with psychiatric patients who do not carry the t(1;11) translocation, to contribute to the elevated risk of major mental illness displayed by t(1:11) translocation carriers^9^.

## Materials and methods

Detailed materials and methods are provided in the Supplementary information file.

## Results

### Adult *Der1* mutant mice show no overt changes in brain structure

Using ex vivo structural MRI, we found no evidence for the effects of the *Der1* mutation on overall brain volume or the volumes of 51 brain regions analysed individually (Supplementary Table 1). In the absence of hypotheses arising from the MRI analysis of brain structure, and given that DISC1 is highly expressed in the hippocampus from early development through to adulthood, and that prefrontal cortex (PFC) is affected in t(1:11) translocation carriers^9^, these regions were explored in further detail. The *Der1* mutation does not affect cell densities in the hippocampal stratum, radiatum, lacunosum and molecular or prefrontal cortex, nor the thickness of individual cortical layers within the barrel cortex, nor the total cortical thickness in either the barrel cortex or the PFC (Supplementary Figs. 2–4).

### RNAseq analysis of adult *Der1* cortex and hippocampus

**Fig. 1:**
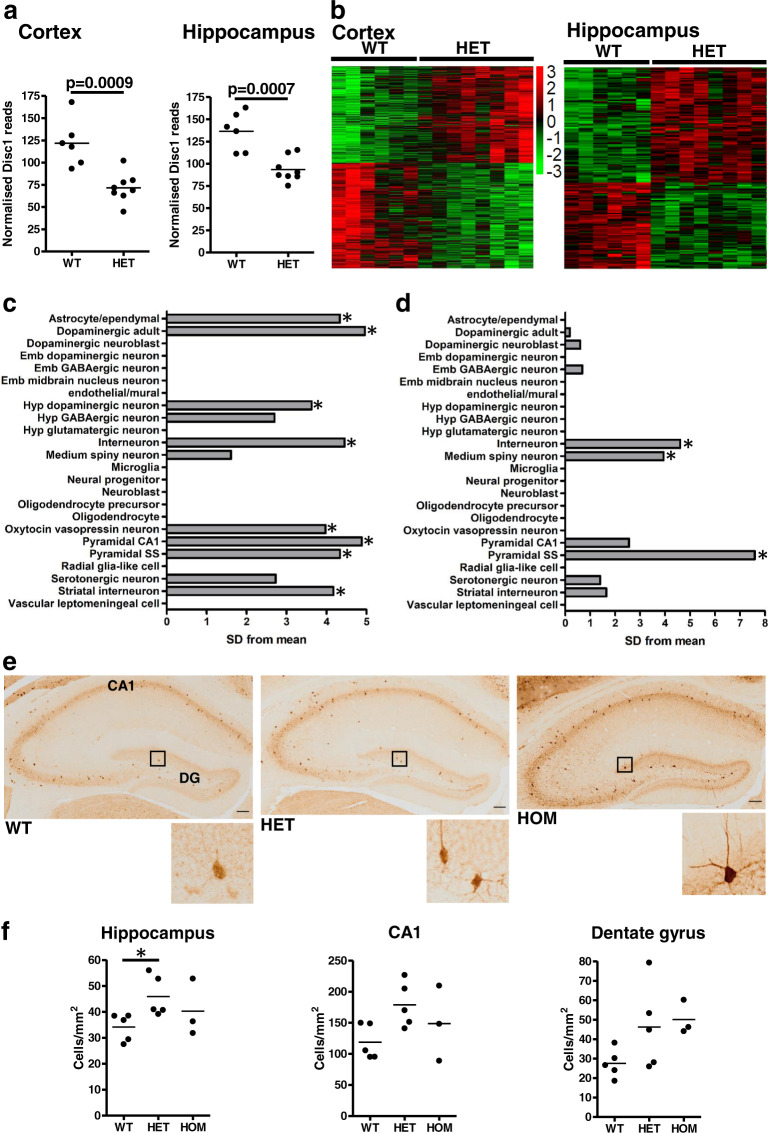
The *Der1* mutation targets specific cell types in heterozygous cortex and hippocampus. **a** *Disc1* RNASeq reads normalised to total reads per sample in wild-type versus heterozygous *Der1* cortex and hippocampus. **b** Heat maps of the top 500 dysregulated genes identified by RNASeq of wild-type versus heterozygous *Der1* cortex and hippocampus. **c**, **d** EWCE analysis of heterozygous *Der1* cortex and hippocampus, respectively, in mouse brain cell classes. Asterisk, significance after Bonferroni correction; Emb embryonic, Hyp hypothalamic, SD standard deviation. **e** Parvalbumin expression in hippocampal sections from 9-week-old mouse brain. Enlarged regions showing parvalbumin-expressing interneurons are indicated by boxes. Scale bars, 100 μm. **f** Average density of parvalbumin-expressing interneurons. Hippocampus refers to the whole hippocampal formation. Data were analysed by Kruskal–Wallis one-way ANOVA, *p* = 0.07 for the dentate gyrus, *p* = 0.049 for the hippocampal formation. The horizontal line on graphs for each sample, an average of values; WT wild type, HET heterozygous *Der1*, HOM homozygous *Der1*, DG dentate gyrus, *p < 0.05.

We next conducted RNASeq using wild-type and heterozygous ‘cortex’ (consisting of cortices minus hippocampus, cerebellum and olfactory bulbs) and hippocampus. The resulting data were analysed at the whole gene and single-exon levels using DESeq2^31^ and DEXSeq^32^, respectively. Full-length Disc1 expression is reduced in heterozygous *Der1* mouse whole brain as detected by a quantitative reverse transcription-polymerase chain reaction and immunoblotting^12^. RNAseq also found reduced *Disc1* expression in heterozygous *Der1* cortex and hippocampus (Fig. 1a, Supplementary Table 2a, c), confirming the validity of these datasets.

Expression of 30,121 genes was detected in the cortex, of which 2124 and 3568 are differentially expressed in heterozygotes at the whole gene or exon level, respectively (all corrected *p* < 0.05, Fig. 1b, Supplementary Table 2a, b). Expression of 28,049 genes was detected in the hippocampus, of which 175 and 52 are differentially expressed in heterozygotes at the whole gene or exon level, respectively (all adjusted *p* < 0.05, Supplementary Table 2c, d).

### Expression-weighted cell-type enrichment (EWCE) analysis of RNASeq data suggests specific cell types are targeted by the *Der1* mutation

EWCE analysis^33^ was used to look for evidence that certain cell types are especially vulnerable to the *Der1* mutation. We utilised gene expression profiles generated by hierarchical clustering of single-cell RNASeq profiles from 9970 mouse brain cells and around 15,000 of the most abundantly expressed genes, resulting in 24 cell classes, referred to as the KI Superset^34^. The authors of that study calculated ‘specificity values’ for each gene within each cell class, to indicate enrichment for expression of that gene in a cell class compared to the other classes in the Superset^34^. EWCE analysis was used here to determine whether there is an enrichment for *Der1*-induced dysregulation of genes with high specificity values for Superset cell classes in the cortex (Fig. 1c). Statistical significance was observed for pyramidal neurons (‘pyramidal somatosensory’, ‘pyramidal CA1’ [which also encompasses neurons from CA2 and the subiculum^34^]), interneurons (‘cortical interneuron’, ‘striatal interneuron’), dopaminergic neurons (‘dopaminergic adult neurons’, ‘hypothalamic dopaminergic neurons’), ‘oxytocin/vasopressin-expressing neurons’ and ‘astrocytes/ependymocytes’. *Der1* hippocampus-dysregulated genes are also highly enriched in several cell classes (Fig. 1d). Of these, pyramidal neurons (‘pyramidal somatosensory’), ‘medium spiny neuron’ and ‘interneuron’ achieved statistical significance. ‘Pyramidal CA1’ reached initial significance in *Der1* hippocampus, but did not survive multiple testing correction. Pyramidal CA1, pyramidal somatosensory and medium spiny neurons should reside primarily in the hippocampus, cortex and striatum, respectively; thus, some of these findings were initially unexpected. However, the previously published hierarchical clustering of cell classes^34^ indicated that ‘pyramidal CA1’ and ‘pyramidal somatosensory’ are highly similar, with medium spiny neurons the next most closely related cell type. We, therefore, infer that in the cortex and hippocampus, the *Der1* mutation may target general features shared between these three neuron classes.

Based on these findings, parvalbumin-expressing interneuron density was quantified in adult PFC and hippocampus. PFC shows no change (Supplementary Fig. 5); however, there is a trend towards an increase in the dentate gyrus (*p* = 0.07), and a significant increase in *Der1* heterozygotes when the whole hippocampus is examined (34% increase, Fig. 1e, f). The EWCE analysis data pointing to hippocampal interneuron targeting could therefore be due, at least partially, to the increased density of interneurons expressing parvalbumin at high levels. This contrasts with previous reports of reduced parvalbumin-positive cell density in mice expressing mutant DISC1 or in response to endogenous Disc1 knockdown^35^.

We also hypothesised that the cell types most affected by the *Der1* mutation might be susceptible to apoptosis, as quantified using activated caspase-3. Of the adult PFC and hippocampal regions examined, there is a trend towards increased apoptosis in CA1 (*p* = 0.06, Supplementary Fig. 6), which may indicate that CA1 cells are particularly vulnerable. This could lead to reduced cell density in CA1, a parameter that unfortunately could not be adequately examined due to the prohibitively tight packing of cells in this region.

### RNASeq deconvolution suggests that cell class proportions are unaltered by the *Der1* mutation

RNASeq deconvolution was carried out utilising gene expression data (rather than the specificity values used above for EWCE analysis) for the most highly enriched genes from the 24 cell classes of the Superset^34^. First, the ability of the deconvolution programme, CIBERSORT^36^, to deconvolve the 24 cell classes was examined by generating artificial in silico samples with varying proportions of each cell class (Supplementary Fig. 7). Using two specificity value thresholds, CIBERSORT was able to deconvolve most cell types. The exceptions include embryonic cell types, which should be absent from our *Der1* samples, and rarer cell types in the adult brain such as neural progenitors and neuroblasts. When used to deconvolve *Der1* cortex and hippocampus whole-gene DESeq2 RNASeq data, CIBERSORT found no evidence for an effect of the mutation upon the relative proportion of any of the cell classes examined (Supplementary Fig. 8). The increased density of hippocampal parvalbumin-positive interneurons (Fig. 1e, f) therefore may not represent a general effect upon all hippocampal interneuron types. Likewise, the trend towards increased hippocampal CA1 apoptosis (Supplementary Fig. 6) does not translate to a detectably decreased density of pyramidal CA1 neurons, possibly because this Superset class also contains pyramidal neurons from CA2 and the subiculum^34^.

### Molecular pathway analysis predicts the wide-ranging effects of the *Der1* mutation

**Fig. 2:**
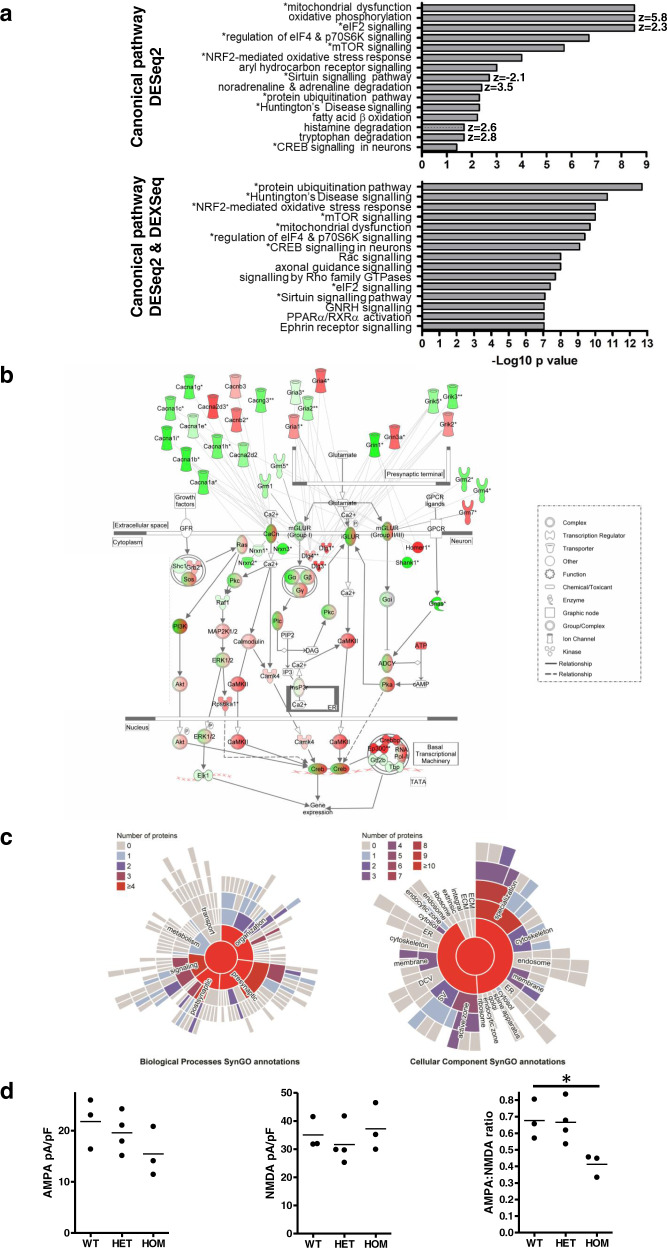
Consequences of the *Der1* mutation. **a** Top relevant canonical pathway predictions for heterozygous *Der1* cortex using whole gene, DESeq2 or whole gene and exon-level, DESeq2 + DEXSeq data. Asterisks indicate pathways highlighted in both cases. Where IPA predicts a direction of change, this is indicated by a *z* score, with positive *z* scores indicating upregulation. **b** Altered gene expression in the ‘CREB signalling in neurons’ canonical pathway in heterozygous *Der1* cortex, determined using the whole gene and exon-level DESeq2+DEXSeq data. To provide additional information, genes encoding calcium channels (CaCh), metabotropic glutamate receptors (mGLUR), ionotropic glutamate receptor subunits (iGLUR) and structural synaptic components have been added to the pathway using the IPA ‘Build’ tool. Transcripts encoding components from the whole pathway are dysregulated at the whole gene and/or isoform level, including ionotropic AMPA and NMDA glutamate receptor subunits, metabotropic glutamate receptors and voltage-gated calcium channels, all of which can control the calcium ion influx or G-protein activation that initiates the pathway. Genes encoding several synaptic scaffolds that are required to generate and maintain synapse structure/size and/or anchor glutamate receptors and calcium channels are also dysregulated, including Shank1, Homer1 and Dlg1/3/4, neurexins and neuroligins. Also dysregulated are genes encoding various factors downstream of glutamate receptors and calcium channels that activate the cAMP-dependent transcription factor CREB, such as various forms of Camk2 and adenylyl cyclases. The transcriptional machinery is additionally affected, including the cAMP-dependent transcription factor complex. Double outlines indicate protein complexes and classes, the components of which can be found in Supplementary Table 2a, b. Colour intensity represents the strength of gene expression change, with graded colour within double-outlined symbols representing the overall direction of change within protein complexes. Green, downregulated; red, upregulated; *genes identified by DEXSeq; ** genes identified by DEXSeq and DESeq2. **c** Sunburst plots showing SynGO- annotated synaptic functions of the dysregulated proteins found in homozygous *Der1* hippocampus synaptosomes (FDR-adjusted *p* value < 0.05). Note that synaptosomes are enriched for the complete presynaptic terminal, the postsynaptic membrane and the postsynaptic density, as well as membranes originating from organelles such as the Golgi and endoplasmic reticulum^73^. **d** Quantification of AMPA and NMDA receptor currents by whole-cell patch clamping of neurons cultured from *Der1* hippocampus. Data were analysed by one-way ANOVA, *p* = 0.03. The horizontal line on graphs for each sample, an average of values; WT wild type, HET heterozygous *Der1*, HOM homozygous *Der1*, *p < 0.05.

Since the patterns of gene dysregulation are not explained by overtly altered cell proportions, the RNASeq data were next used to predict the effects upon canonical pathways. Ingenuity pathway analysis (IPA), an unbiased method for examining transcriptomic data using statistical significance and magnitude plus direction of fold change, was carried out using the whole-gene level DESeq2 data, or combined DESeq2 plus exon-level DEXSeq data. This analysis predicts the effects upon diverse pathways, including metabolic, stress–response and important neuro signalling processes (Fig. 2a). A selection of these pathways, based on statistical significance or relevance to later parts of this study, is discussed below.

Mitochondrial dysfunction, including increased oxidative phosphorylation, is strongly predicted, based largely on the upregulated whole-gene expression of multiple complexes I, III and IV components (Supplementary Fig. 9), consistent with DISC1’s known role in regulating oxidative phosphorylation^37,38^. Moreover, the chimeric transcripts expressed by *Der1* mice encode aberrant mitochondrial species that induce mitochondrial dysfunction^29^.

Also upregulated at the whole gene level is the mitochondrial pathway ‘Fatty acid β-oxidation I’ (Supplementary Fig. 10), which degrades fatty acids to release energy. Dysregulated enzymes feeding into this pathway are involved in the fatty acid synthesis and break down. Together, these changes imply altered levels of lipids, which are critical for many brain processes.

The ‘CREB signalling in neurons’ pathway (Fig. 2b) is activated by cell surface glutamate receptors, including AMPA and NMDA receptors, and calcium channels. It regulates gene expression changes that are critical for synaptic plasticity and long-term potentiation (LTP), both known to be DISC1-modulated^30^. DISC1 is also already known to regulate CREB signalling^30^, and in our study, IPA predicts that downregulation of Creb1 activity is responsible for many of the gene expression changes (*p* = 9e−9, *z* = −3). Indeed, there is enrichment (hypergeometric *p* = 0.02) for dysregulation of genes containing conserved cAMP-response elements (CREs, http://natural.salk.edu/creb/, Supplementary Table 3) in heterozygous *Der1* cortex, with 203 (9.6%) of the genes dysregulated at the whole-gene level having CREs. The *Der1* mutation potentially affects the activation of the pathway via AMPA receptor subunit degradation^39^, and NMDA receptor membrane dynamics and surface expression^12^. Moreover, genes encoding glutamate receptors, including AMPA and NMDA receptor subunits, and several synaptic scaffolds are dysregulated (Fig. 2b).

Using the combined dysregulated DESeq2 plus DEXSeq data, IPA also determined that many cellular functions are enriched for differentially expressed genes (Table 1, Supplementary Table 4a). Predictions relating to neurotransmission, synaptic plasticity and LTP are related to the ‘CREB signalling in neurons’ pathway above, plus genes encoding inhibitory signalling factors, such as subunits of GABA_A_ and GABA_B_ receptors. Predictions relating to vesicle transport and exo-/endocytosis are based on dysregulated genes encoding vesicle-trafficking factors, voltage-gated calcium channel subunits as well as synaptotagmins and syntaxins that together mediate calcium-dependent neurotransmitter release, components of the exocyst complex and components of the endocytic Clathrin-associated adaptor–protein complex. The wide-ranging neuronal morphology and cytoskeleton-related predictions are based on multiple genes involved in diverse relevant processes. Similarly, the cell–cell contact/adhesion-related functions are widespread, but notably encompass genes required for early synapse formation, such as latrophilins, as well as maintenance of trans-synaptic connections, for example, neuroligins and neurexins. Other predictions relate to cell proliferation, neuronal migration and circadian rhythms. All of these processes are already known to involve DISC1^12,16,18,21,25,35,40–43^.

**Table 1:** Top relevant altered cellular functions in heterozygous *Der1* mouse cortex and human iPSC-derived neurons from members of the t(1:11) translocation family predicted using DESeq2 + DEXSeq data. All *Der1* mouse and human t(1;11) neuron functions are listed in Supplementary Table 4.

Function (no. of molecules^a^)	*Der1* cortex score (no. of genes^b^)	Human t(1:11) translocation neuron culture score (no. of genes^b^)	Hypergeometric *p* value for enrichment (no. of shared genes^c^)
*General cell morphology*			
Development of neurons (1423)	*p* = 2e−53 (457)	*p* = 2e−13 (148)	***p*** = **1e−3** (63)
Morphogenesis of neurons (1080)	*p* = 1e−47 (360)	*p* = 4e−13 (119)	***p*** = **3e−4** (56)
Morphology of neurons (1123)	*p* = 7e−37 (303)	*p* = 1e−4 (80)	***p*** = **6e−5** (37)
Morphology of cells (4370)	*p* = 1e−29 (902)		
Abnormal morphology of neurons (923)	*p* = 6e−25 (212)		
*Cell contact*			
Cell–cell contact (1,118)	*p* = 5e−26 (299)	*p* = 4e−6 (92)	***p*** = **6e−4** (38)
Development of gap junctions (327)	*p* = 1e−18 (123)	*p* = 2e−4 (37)	*p* = *0.08* (17)
Formation of cell–cell contacts (414)	*p* = 6e−16 (138)	*p* = 8e−6 (48)	*p* = *0.08* (19)
Formation of intercellular junctions (409)	*p* = 1e−15 (136)	*p* = 1e−5 (47)	*p* = *0.07* (19)
Formation of plasma membrane (406)	*p* = 1e−15 (134)	p = 3e−6 (48)	*p* = *0.05* (20)
*Cytoskeleton*			
Organisation of cytoplasm (2,832)	*p* = 2e−64 (791)	*p* = 3e−16 (257)	***p*** = **2e−6** (104)
Organisation of cytoskeleton (2,624)	*p* = 4e−57 (720)	*p* = 3e−16 (240)	***p*** = **1e−8** (101)
Microtubule dynamics (2247)	*p* = 6e−54 (627)	*p* = 1e−14 (206)	***p*** = **1e−6** (87)
Development of cytoplasm (873)	*p* = 9e−19 (233)	*p* = 2e−4 (71)	***p*** = **1e−5** (35)
Formation of cytoskeleton (733)	*p* = 5e−14 (179)		
*Cellular protrusions/neurites*			
Neuritogenesis (1067)	*p* = 2e−46 (354)	*p* = 4e−12 (115)	***p*** = **2e−4** (55)
Formation of cellular protrusions (1645)	*p* = 1e−46 (488)	*p* = 3e−15 (170)	***p*** = **2e−4** (70)
Growth of neurites (910)	*p* = 5e−30 (261)	*p* = 1e−7 (81)	***p*** = **6e−4** (36)
Morphology of cellular protrusions (522)	*p* = 3e−25 (166)		
Morphology of neurites (414)	*p* = 6e−25 (139)		
*Axons*			
Axonogenesis (338)	*p* = 1e−18 (122)	*p* = 2e−7 (45)	***p*** = **2e−3** (25)
Morphology of axons (169)	*p* = 2e−16 (65)		
Growth of axons (281)	*p* = 2e−12 (87)		
Abnormal morphology of axons (133)	*p* = 4e−11 (46)		
Guidance of axons (202)	*p* = 5e−10 (71)	*p* = 2e−5 (29)	ns (12)
*Dendrites*			
Formation of dendrites (209)	*p* = 9e−19 (90)		
Dendritic growth/branching (446)	*p* = 8e−18 (131)		
Density of dendritic spines (143)	*p* = 1e−11 (49)		
Morphology of dendrites (138)	*p* = 3e−9 (49)		
Abnormal morphology of dendrites (75)	*p* = 3e−8 (32)		
*Cell proliferation*			
Proliferation of neuronal cells (1066)	*p* = 5e−28 (290)	*p* = 2e−9 (98)	***p*** = **2e−3** (39)
*Neuronal migration*			
Migration of neurons (362)	*p* = 1e−16 (125)	*p* = 8e−6 (43)	***p*** = **0.03** (20)
*Circadian rhythm*			
Circadian rhythm (132)	*p* = 3e−8 (55)		
*Transport*			
Organisation of organelle (948)	*p* = 1e−23 (270)		
Transport of vesicles (192)	*p* = 1e−14 (69)		
Endocytosis (924)	*p* = 8e−10 (202)	*p* = 3e−6 (76)	***p*** = **2e−3** (27)
Secretory pathway (367)	*p* = 8e−10 (93)		
Formation of vesicles (307)	*p* = 4e−9 (70)		
*Neurotransmission*			
Neurotransmission (716)	*p* = 5e−31 (233)	*p* = 5e−5 (62)	***p*** = **0.03** (26)
Potentiation of synapse (546)	*p* = 1e−28 (165)		
Long-term potentiation (539)	*p* = 4e−28 (163)		
Synaptic transmission (558)	*p* = 4e−27 (191)	*p* = 7e−06 (55)	***p*** = **0.04** (24)
Developmental process of synapse (303)	*p* = 2e−18 (117)	*p* = 1e−4 (36)	*p* = *0.08* (17)
Excitatory postsynaptic potential (166)	*p* = 2e−15 (72)		
Long-term potentiation of brain (281)	*p* = 2e−13 (74)		
Plasticity of synapse (170)	*p* = 2e−12 (66)		
Long-term potentiation of cerebral cortex (254)	*p* = 6e−12 (66)		
Miniature excitatory postsynaptic currents (71)	*p* = 1e−11 (38)		

A full list of functions is provided in Supplementary Table 4a, d. Related functions are grouped, with top functions shown for each group. (a) a total number of molecules relating to each IPA function; (b) number of dysregulated genes relating to each function; (c) a number of genes relating to function that are dysregulated in both *Der1* cortex and human t(1:11) translocation neurons; italics, trend; *ns* not significant.

*p* values that are statistically significant are shown in bold.

*Der1* hippocampus RNASeq data were similarly analysed. IPA did not strongly predict any canonical pathway changes due to the relatively small number of changes but did predict altered functions that largely reflect those for the cortex (Supplementary Tables 4b and 5), and there is an enrichment for dysregulation of 86 shared genes (39% of the total dysregulated hippocampal genes, Supplementary Table 2c, d) in both regions (*p* = 7e−11). Myelination is also predicted to be affected, consistent with previous studies demonstrating DISC1 involvement in oligodendrocyte differentiation and function^44–46^.

Numerous processes are thus predicted to be affected by the *Der1* mutation in the cortex and hippocampus, with striking convergence upon neurotransmission.

### Molecular pathway analysis of targeted cell types identified by EWCE analysis

EWCE analysis identified cell classes that may be targeted by the *Der1* mutation (Fig. 1c, d). We reasoned that the cell class-enriched gene expression changes may inform on the impact of the *Der1* mutation in each cell type. Pathway analysis was therefore carried out using the cell class-enriched dysregulated genes (Supplementary Tables 6 and 4c). *Der1* cortex pyramidal neuron (CA1 and somatosensory) and interneuron terms relate to synaptic transmission. *Der1* cortex astrocyte/ependymocyte terms relate to lipid metabolism and uptake of glutamine/glutamate. The lipid metabolism predictions are based on upregulation of genes encoding enzymes involved in fatty acid β-oxidation, and other aspects of brain lipid metabolism. This is related to the *Der1* cortex RNASeq canonical pathway prediction ‘Fatty Acid β-oxidation I’ (Fig. 2a), and indicates a potential imbalance between lipid synthesis and oxidation. Since astrocytes are a major source of brain lipid that is widely utilised, including for synapse function^47^ and myelination by oligodendrocytes^48^, these processes may be compromised via astrocyte dysfunction. The glutamine/glutamate uptake predictions are based on the dysregulated expression of genes such as *Slc1a2*, which encodes the synaptic glutamate transporter Eaat2. Astrocytes are critical regulators of glutamine and glutamate homoeostasis in the brain, which includes glutamate clearance from synapses, and consequent regulation of glutamatergic neurotransmission and synaptic plasticity^49^. There were no convincing findings for the other cell classes examined.

### Shared gene dysregulation in heterozygous *Der1* cortex and t(1:11) translocation carrier-derived cortical neuron cultures confirms the relevance of the *Der1* RNASeq findings to psychiatric illness

To determine how the above RNASeq data analyses of the *Der1* mouse relate to the t(1:11) translocation, we compared the *Der1* mouse data to previously published RNASeq data generated from t(1:11) translocation carrier-derived neuron cultures^12^. Human iPSC-derived neurons grown in culture are not directly comparable to adult mouse brain tissue. Even so, a trend towards enrichment for shared gene expression changes was evident from the 20 dysregulated genes in common between IPSC-derived cortical neuron cultures from t(1:11) translocation carriers^12^ and heterozygous *Der1* hippocampus (*p* = 0.06, Supplementary Table 2c, d), while 511 genes are differentially expressed in both heterozygous *Der1* mouse cortex and the IPSC-derived cortical neuron cultures (Supplementary Table 2a, b), demonstrating significant enrichment (*p* = 1e−14), and further validating the *Der1* mouse as an accurate model for the effect of the t(1:11) translocation upon DISC1 expression. An overlapping set of cellular functions were also identified in the human cortical neuron cultures and heterozygous *Der1* mouse cortex (Table 1, Supplementary Table 4a, d). Moreover, for most of the shared functions, there is either significant enrichment or a trend towards enrichment for a common set of differentially expressed genes (Table 1). This convergence indicates that disruption of *DISC1* likely contributes substantially to the altered molecular pathways in the human neuron cultures.

Nonetheless, several functions are enriched in the *Der1* cortex data, but not in the human cortical neuron data. Many relate specifically to synaptic plasticity and LTP, processes that are constitutive in the brain, but which require stimulation to be detected in neuronal cultures. A number of other changes relate specifically to the development of dendrites, which may not reach maturity in IPSC-derived neuronal cultures^50^.

### Mass spectrometry and SynGO analysis of adult *Der1* synaptosomes confirm synaptic changes

To complement the RNASeq analysis, synaptosome fractions were prepared from hetero- or homozygous *Der1* mice and mass spectrometry was used to determine whether synaptosomal protein expression profiles differ between mutant and wild-type mice. Of the 2783 detected proteins in the cortex, no changes survived multiple-correction testing in synaptosomes prepared from *Der1* mice (Supplementary Table 7a, Supplementary Fig. 11). Of the 2183 proteins detected in the hippocampus, 62 were found to be dysregulated in homozygotes (false-discovery rate (FDR)-adjusted *p* value < 0.05, Supplementary Table 7b, Supplementary Fig. 11). These proteins were annotated to well-established synaptic genes using the SynGO database^51^ (Fig. 2c, Supplementary Table 7c). This is an expert-curated database of gene ontology terms relating to synapses. From the 62 regulated proteins, 26 were found annotated in SynGO, 24 with cellular component annotation and 19 with biological process annotation. Dysregulated proteins were found annotated across a wide spectrum of pre- and post-synapse functions. For instance, several proteins were annotated to the postsynaptic density, such as Camk2a (downregulated), AMPA receptor subunits (downregulated), the DISC1 interactor Trio^52^, which modulates AMPA receptor currents in hippocampal CA1 pyramidal neurons^53^, vesicle protein Exoc4, an exocyst component and the SNARE STX7, and Gad2, a presynaptic protein that synthesises GABA in interneurons. These changes point to the effects upon similar synaptic processes to those highlighted by RNASeq analysis. However, fewer changes were detected in the synaptosomes, probably due to the lower number of proteins identified in comparison with the RNASeq analysis, in which many relevant RNASeq changes were detected at the isoform level.

### Functional effects of the *Der1* mutation upon synapses

The RNAseq data point towards the effects of the *Der1* mutation upon synapses, which was confirmed by subsequent synaptic proteomics analysis. The observed changes include subtly altered expression of NMDA receptor isoforms and reduced AMPA receptor subunit levels. Moreover, we previously demonstrated that cultured hippocampal neuron dynamics and cell surface/synaptic expression of NMDA receptors are dysregulated by the *Der1* mutation^12^.

To examine these receptors functionally, whole-cell patch-clamping was used to record currents from both receptor types in mature cultured hippocampal neurons (Fig. 2d). The AMPA:NMDA ratio is decreased in homozygous *Der1* neurons indicating a functional imbalance between these two receptor subtypes. This may be due in part to altered AMPA receptor currents, which although not statistically significant, are decreased in hetero- and homozygous neurons. To discover whether this whole-cell patch-clamp finding extends to receptors located at synapses in heterozygous *Der1* hippocampus, and in the cortex, will require future in-depth electrophysiological measurements. If it does indeed extend to synapses, the decreased AMPA:NMDA ratio could have many consequences, including impaired triggering of NMDA receptor-dependent LTP, which is initiated by AMPA receptor-induced release of the magnesium block on NMDA receptors.

### Enrichment for dysregulation of putative schizophrenia, bipolar disorder and depression risk genes in heterozygous *Der1* cortex and hippocampus

**Fig. 3:**
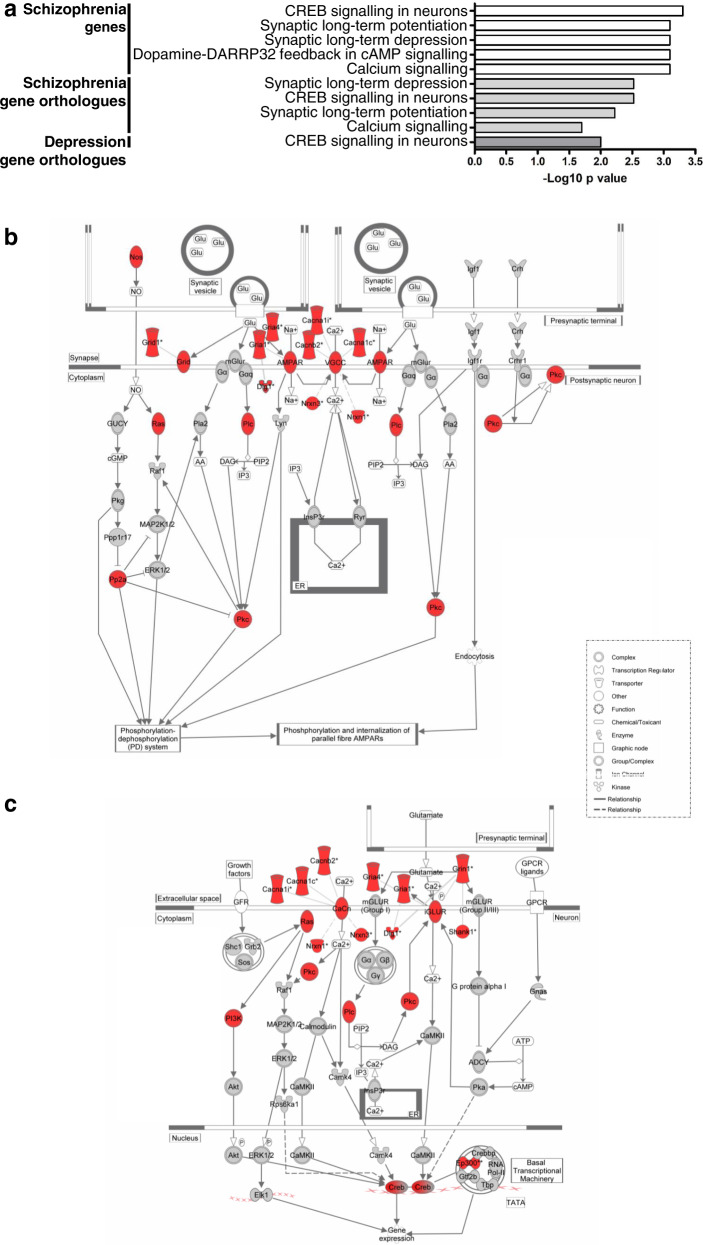
The *Der1* mutation dysregulates canonical pathways and genes related to schizophrenia and depression in heterozygous *Der1* cortex. **a** Canonical pathway predictions for putative schizophrenia risk genes, and for orthologues of putative schizophrenia and depression risk genes that are dysregulated at the whole-gene and exon-level, as identified using DESeq2 + DEXSeq data. **b**, **c** Altered schizophrenia risk gene orthologue expression in the ‘Synaptic long-term depression’ and ‘CREB signalling in neurons’ canonical pathways, respectively. Double outlines indicate protein complexes and classes, the components of which can be found in Supplementary Table 2a, b. To provide additional information, genes encoding ionotropic glutamate receptor *δ* subunits (Grid), AMPA receptor subunits (AMPAR), voltage-gated calcium channel subunits (VGCC), calcium channels (CaCh), ionotropic glutamate receptor subunits (iGLUR) and structural synaptic components have been added to the pathways using the IPA ‘Build’ tool. *Genes identified by DEXSeq; red, dysregulated putative schizophrenia risk gene orthologue.

A large number of putative schizophrenia risk genes have been identified from two large-scale GWAS and one large-scale CNV study^1–3^. IPA maps many of these genes to shared molecular pathways. The top canonical pathway (Fig. 3a) is ‘CREB signalling in neurons’. Others include ‘Synaptic long-term potentiation’ and ‘Synaptic long-term depression’, both mechanisms underlying synaptic plasticity. These findings largely agree with previous observations^54^.

The heterozygous *Der1* cortex-combined RNASeq DESeq2 plus DEXSeq data were compared to the list of putative schizophrenia risk genes used above^1,2^, but including only genes encoding synaptic proteins from the CNV study^3^ as defined by its authors. This identified significant enrichment for dysregulation of schizophrenia candidate gene orthologues (Table 2, Supplementary Table 2a, b). The top canonical pathways identified using this set of genes for IPA are ‘Synaptic long-term depression’, ‘CREB signalling in neurons’, ‘Synaptic long-term potentiation’ and ‘Calcium signalling’ (Fig. 3a–c). These predictions are among the top five of those obtained using the full set of putative schizophrenia risk genes (Fig. 3a), indicating that the *Der1* mutation and genetic risk factors for schizophrenia converge upon the same pathways.

**Table 2:** Enrichment for dysregulated expression of putative psychiatric illness risk gene orthologues in *Der1* cortex and hippocampus.

Study	Loci	Genes	Dysregulated in cortex	Hypergeometric *p* value for enrichment in the cortex	Dysregulated in hippocampus	Hypergeometric *p* value for enrichment in the hippocampus
GWAS, schizophrenia^1^	108	348	121 genes at 61 loci	***p*** = **1e−13** (***p*** = **8e−19**)	6 genes at 6 loci	***p*** = **0.04** (***p*** = **2e−4**)
GWAS, schizophrenia^2^	143	481	127 genes at 73 loci	***p*** = **3e−6** (***p*** = **8e−19**)	6 genes at 6 loci	*p* = *0.09* (***p*** = **8e−4**)
MAGMA, schizophrenia^2^		535	210	***p*** = **3e−30**	15	***p*** = **2e−5**
CNV (synapse genes), schizophrenia^3^		52	25	***p*** = **8e−7**	4	***p*** = **7e−4**
GWAS, depression^4^	44	70	19 at 19 loci	***p*** = **0.02** (***p*** = **9e−5**)	1	ns
MAGMA, depression^4^		153	33	***p*** = **0.047**	1	ns
MAGMA meta-analysis, depression^6^		269	94	***p*** = **4e−11**	6	***p*** = **0.01**
GWAS, bipolar disorder^5^	30	218	73 at 21 loci	***p*** = **4e−8** (***p*** = **8e−10**)	3 genes at 3 loci	ns (***p*** = **2e−3**)
MAGMA, bipolar disorder^5^		152	49	***p*** = **2e−5**	3	*p* = *0.09*
GWAS, Alzheimer’s Disease^55^	21	102	9 at 9 loci	***p*** = **3e−3 (*p*** = **6e−3)**	1	ns
GWAS cerebral cortex architecture^65^		193	57	***p*** = **5e−5**	1	ns

Loci indicate the number of associated genomic loci identified by GWAS. Genes indicate the total number of genes at the associated loci or the total number identified by MAGMA. Bracketed *p* values indicate enrichment for loci containing at least one dysregulated gene orthologue. italics, trend; *ns* not significant.

*p* values that are statistically significant are shown in bold.

Enrichment for dysregulation of schizophrenia candidate gene orthologues was also apparent using the heterozygous *Der1* hippocampus-combined RNAseq DESeq2 plus DEXSeq data (Table 2, Supplementary Table 2c, d), although there were too few genes to carry out meaningful pathway analysis.

Large-scale genetic data are also available for bipolar disorder and depression^4–6^. IPA did not find that the genes identified from these studies converge strongly upon any canonical pathways, although a subset of depression-associated genes is involved in synaptic structure and activity^6^. Nonetheless, there is an enrichment for dysregulation of the orthologues of candidate genes for both disorders in *Der1* cortex, and for depression in *Der1* hippocampus (Table 2, Supplementary Table 2). Moreover, the dysregulated putative depression risk gene orthologues in *Der1* cortex predict the effects upon the ‘CREB signalling in neurons’ pathway (Fig. 3a).

We also examined overlaps between genes dysregulated in the *Der1* mouse and two non-psychiatric illness-related large-scale GWAS. For Alzheimer’s disease^55^, there is an enrichment for dysregulation of candidate gene orthologues in *Der1* cortex (Table 2, Supplementary Table 2), with six of the nine gene matches (ABCA7, APOE, CLU, FERMT2, PTK2B/PYK2 and SORL1) involved in amyloid-beta (Aβ)-related processes^56–61^. This effect may be explained by observations that DISC1 interacts with amyloid precursor protein^62^, and regulates Aβ generation^63,64^. The second comparison was to a study of cerebral cortex architecture^65^. Again, there is an enrichment for dysregulation of candidate gene orthologues in *Der1* cortex (Table 2, Supplementary Table 2), although no molecular pathways are highlighted.

The enrichment for dysregulation of orthologues of candidate genes for brain disorders (which is particularly striking for schizophrenia) when combined with convergence upon specific molecular pathways already implicated in those disorders, indicates that the *Der1* mutation may exert effects that are directly relevant to these human brain illnesses.

## Discussion

Heterozygous *Der1* mutant mice accurately recapitulate the effects of the t(1:11) translocation upon DISC1 expression in IPSC-derived neural precursors and cortical neurons^12^. We now demonstrate that patterns of gene expression dysregulation and pathway predictions are similar between heterozygous *Der1* cortex and IPSC-derived cortical neuronal cultures from t(1:11) translocation carriers. Together, these observations suggest that *DISC1* disruption is an important factor in the increased risk of major mental illness displayed by t(1:11) translocation carriers, and argue that the *Der1* mouse model can be used to study the neuronal effects of *DISC1* disruption upon brain function to understand disease mechanisms in these individuals.

Many of the findings reported here are consistent with known DISC1 biology and brain function, but observations such as the lack of overt brain structural changes, and of increased density of hippocampal parvalbumin-expressing interneurons, were unexpected on the basis of previously described DISC1 mutant mice that model aspects of the effects of the t(1;11) translocation upon DISC1 expression^35^ (Supplementary Table 8). Such differences, and the many phenotypic differences between previously published mutants (Supplementary Table 8), accentuate the critical importance of studying a mutant that accurately mimics all effects of the t(1;11) translocation in order to understand disease mechanisms in t(1;11) translocation carriers. Other findings, such as the predicted dysregulation of astrocyte lipid metabolism, have not been reported previously. This is the first, and only, mutant mouse to accurately model the effects of the t(1:11) translocation, and it, therefore, provides important and new insights into molecular mechanisms underlying the increased disease risk and psychiatric symptoms of t(1:11) translocation carriers.

Structural and functional brain abnormalities have been reported in human t(1:11) translocation carriers^11^, whereas none were detected in the adult *Der1* mice studied here. This difference may reflect fundamental species differences in brain structure and development, and/or secondary genetic or environmental factors consequent upon, or interacting with, the t(1:11) translocation event. Genetic effects may include loss of normal function of the additional disrupted genes *DISC2* and *DISC1FP1*^13,29^, or an influence of genetic modifiers^28^. Environmental effects may include greater relative age and duration of chronic mental illness with associated long-term exposure to medication such as antipsychotic drugs. The latter progressively decreases grey matter volume in schizophrenia patients^66^ and decreases cortical volume in rats^67^.

The absence of brain structural changes, together with the lack of evidence for altered cell class proportions from RNASeq data deconvolution, indicates that the subtle transcriptomic and proteomic alterations identified in the *Der1* mouse are principally due to altered cellular properties that are largely conserved between it and t(1:11) translocation carriers. EWCE analysis of RNASeq data suggests that the *Der1* mutation may target distinct cell types, including pyramidal neurons (CA1 and somatosensory) and interneurons. These findings correlate well with a previous EWCE analysis using large-scale schizophrenia GWAS data^2,34^ which found that schizophrenia-associated SNPs map to genomic loci containing genes that are highly expressed in a limited number of brain cell types, including CA1 and somatosensory pyramidal neurons and interneurons^34^, thus implicating these cell types in the aetiology of schizophrenia. The additional cell types that appear to be targeted by the *Der1* mutation: dopaminergic neurons, oxytocin/vasopressin-expressing neurons and astrocytes/ependymocytes, were not implicated in schizophrenia by the genomic EWCE analysis. However, dopamine signalling is heavily implicated in schizophrenia, in part because all antipsychotic drugs in clinical use target the dopamine D2 receptor^68^, while *DRD2* is located at a genetic locus repeatedly found to associate with schizophrenia^1,2^ and also with depression^6^. The neuropeptides oxytocin and vasopressin regulate many processes, including social behaviour and anxiety^69^, and are widely implicated in psychiatric disorders^70^. Astrocyte abnormalities have also been reported in relation to psychiatric disorders^71^. Thus, even if not directly targeted by genomic risk variants, these additional cell types do apparently contribute to psychiatric illness.

Pyramidal neurons are the major excitatory neurons in the brain. Interneurons are inhibitory and regulate neuronal network excitability, primarily of pyramidal neurons. Our analyses suggest widespread targeting of pyramidal neurons and interneurons by the *Der1* mutation; thus, excitation and inhibitory control of neuronal networks may be impaired. Neuronal activity could be further impaired if the EWCE predictions are correct and glutamate uptake by astrocytes is dysregulated. Our findings and predictions relating to pyramidal neurons, which are glutamatergic cells, and to astrocytic glutamate uptake, may be related to the decreased glutamate levels detected by brain imaging of t(1:11) translocation carriers^9^. Altered lipid production by astrocytes may be an additional factor affecting neuronal activity. Lipids are required for many processes, including synaptic activity^47^ and myelination^48^. We have previously demonstrated impaired myelination in *Der1* cortex, which is presumably due, at least partially, to direct effects of the mutation upon oligodendrocytes because the corresponding IPSC-derived oligodendrocytes from t(1:11) translocation carriers are abnormal^46^. EWCE analysis did not, however, find evidence that oligodendrocytes are strongly targeted by the *Der1* mutation, although some genes highly specific for this cell type are dysregulated, such as myelin–oligodendrocyte–glycoprotein in the cortex (Supplementary Table 2a), while genes that impact myelination are dysregulated in the hippocampus (Supplementary Tables 4b and 5). Altered lipid production by astrocytes could therefore be a contributory factor in the myelination phenotype.

Consistent with the targeting of cell types implicated in schizophrenia, the *Der1* mutation dysregulates orthologues of many genes implicated as risk factors for schizophrenia and depressive disorders through large-scale GWAS and CNV studies, as we have previously shown for the t(1:11) translocation in IPSC-derived neurons^12^. The pathways by which the t(1:11) translocation causes the major mental illness may therefore overlap those targeted by common genetic risk factors for schizophrenia and depression. We speculate that disruption of the gene encoding the molecular scaffold DISC1, with knock-on effects for its numerous binding partners and functions can, at least partially, recapitulate the consequences of the more common scenario in psychiatric patients whereby multiple interacting common genetic risk factors are inherited, with both scenarios converging upon the same biological pathways. In agreement with this, the symptoms of t(1:11) translocation carriers are indistinguishable from the typical spectrum of clinical presentation of the psychiatric disorders with which they are diagnosed.

The convergence of the *Der1* mutation with a subset of putative common genetic risk factors for schizophrenia and depressive disorders, and the convergence of this subset of genes upon synapses and synaptic plasticity^6,54^ implies that, of all the *Der1* cortex pathway predictions, dysregulated neurotransmission and synaptic plasticity are among the most critical factors in the psychiatric symptoms of t(1:11) translocation carriers. Notably, synaptic plasticity underpins cognition, which is characteristically impaired in schizophrenia.

Altogether, the EWCE and pathway analyses pointing to potential pyramidal neuron and interneuron dysfunction in the hippocampus, the evidence that the number of apoptotic cells in CA1 may be increased, the higher density of hippocampal parvalbumin-positive interneurons, the extensive changes to synaptic protein expression in hippocampus synaptosomes and the electrophysiology data indicating reduced AMPA:NMDA ratio in cultured hippocampal neurons, suggest that hippocampal circuits are especially sensitive to the mutation, although the effects upon other brain regions are also likely.

The hippocampus has multiple input/output pathways from/to other brain regions that are regulated by various neurotransmitters. Hippocampal dysfunction in *Der1* mice could thus have numerous extrinsic/intrinsic causes and knock-on effects. CA1 pyramidal neurons provide the major hippocampal output, including the hippocampal-to-PFC pathway that regulates NMDA receptor-dependent LTP and cognition^72^. This pathway is widely implicated in psychiatric disorders^72^. It is thus an exemplar of the mechanisms by which *DISC1* disruption could confer susceptibility to major mental illness by bringing together the diverse effects described here and elsewhere^12,46^, in our studies of neural cells derived from t(1:11) translocation carriers, and of the corresponding *Der1* mouse. Our findings thus provide important insights into potential disease mechanisms involving specific molecular pathways/functions and cell types in t(1:11) translocation carriers that are likely relevant to schizophrenia and affective disorders in general.

## Supplementary information

Bonneau et al. Supplementary material

Supplementary Table 1

Supplementary Table 2

Supplementary Table 3

Supplementary Table 4

Supplementary Table 7
