# Supplementary material for: Functional brain defects in a mouse model of a chromosomal t(1;11) translocation that disrupts DISC1 and confers increased risk of psychiatric illness
Source: Transl Psychiatry. 2021 Feb 19;11:135. doi: 10.1038/s41398-021-01256-3 (PMC7895946; doi:10.1038/s41398-021-01256-3)
Supplement: Supplementary file 1 — Bonneau et al. Supplementary material [file 41398_2021_1256_MOESM1_ESM.docx]

**Functional brain defects in a mouse model of a chromosomal t(1;11) translocation that disrupts *DISC1* and confers increased risk of psychiatric illness**

**Supplementary information**

Marion Bonneau^1*^, Shane T. O’Sullivan^1^, Miguel A. Gonzalez-Lozano^2^, Paul Baxter^3, 4^, Phillippe Gautier^5^, Elena Marchisella^6^, Neil R. Hardingham^7^, Robert A. Chesters^8^, Helen Torrance^1^, David M. Howard^9, 10^, Maurits A. Jansen^11^, Melanie McMillan^12^, Yasmin Singh^13^, Michel Didier^14^, Frank Koopmans^2^, Colin A. Semple^5^, Andrew M. McIntosh^10^, Hansjürgen Volkmer^15^, Maarten Loos^6^, Kevin Fox^7^, Giles E. Hardingham^3, 4^, Anthony C. Vernon^8, 16^, David J. Porteous^1^, August B. Smit^2^, David J. Price^3^, J. Kirsty Millar^1^

^1^Centre for Genomic and Experimental Medicine, MRC Institute of Genetics and Molecular Medicine at the University of Edinburgh, Edinburgh, UK

^2^Department of Molecular and Cellular Neurobiology, Center for Neurogenomics and Cognitive Research, VU University, Amsterdam, The Netherlands

^3^Centre for Discovery Brain Sciences, Hugh Robson Building, The University of Edinburgh, Edinburgh, UK

^4^UK Dementia Research Institute, Edinburgh Medical School, The University of Edinburgh, Edinburgh, UK

^5^MRC Human Genetics Unit, MRC Institute of Genetics and Molecular Medicine at the University of Edinburgh, Edinburgh, UK

^6^Sylics Synaptologics BV, Amsterdam, The Netherlands

^7^School of Biosciences, Museum Avenue, Cardiff University, Cardiff, UK

^8^Department of Basic and Clinical Neuroscience, Institute of Psychiatry, Psychology and Neuroscience, King’s College London, London, UK

^9^Social, Genetic and Developmental Psychiatry Centre, Institute of Psychiatry, Psychology & Neuroscience, King's College London, UK

^10^Division of Psychiatry, Kennedy Tower, The University of Edinburgh, Edinburgh, UK

^11^Edinburgh Preclinical Imaging, The Chancellor’s Building, The University of Edinburgh, Edinburgh, UK,

^12^Centre for Reproductive Health, The Queen’s Medical Research Institute, The University of Edinburgh, Edinburgh, UK

^13^Centre for Genomics and Transcriptomics, Paul-Ehrlich-Straße 23, Tübingen, Germany

^14^Translational Sciences at Sanofi, Chilly-Mazarin, France

^15^Department of Molecular Biology, NMI Natural and Medical Sciences Institute at the University of Tübingen, Reutlingen, Germany

^16^MRC Centre for Neurodevelopmental Disorders, King’s College London, London, UK

*Corresponding author

Marion Bonneau, now at Washington University in St. Louis, M.O, USA

Tel: +1 314 3332238

Email: [bonneau.mb@gmail.com](mailto:bonneau.mb@gmail.com)

Address: Department of Biology, Washington university, CB 1137, 1 Brookings Drive, St. Louis, M.O, 63130-4899

**Materials and methods**

## Mouse colony maintenance

Mice were housed in the Biomedical Research Facility at the University of Edinburgh. All mice were maintained in accordance with Home Office regulations, and all protocols were approved by the local ethics committee of the University of Edinburgh. Mouse genotyping was carried out as previously described^1^.

## Perfusion fixation and brain isolation

## Mice were anaesthetized with intraperitoneal injection of 0.1ml/10g Fentanyl/Fluanisone (Hypnorm®) and Midazolam (Hyponovel®). Deep anaesthesia was ensured by measuring withdrawal reflexes. The mice were then transcardially perfused with 4% paraformaldehyde at a rate of 0.2-0.5 ml/second. Brains were dissected out and the olfactory bulbs and cerebellum removed. Brains were transferred to 4% neutral buffered formalin for 24 hr, then stored in 70% ethanol.

## Magnetic resonance imaging

Brains were taken from twelve same-sex littermate genotype trios (one wild-type, one heterozygote, one homozygote from the same litter, six male and six female trios). Brains were removed from 70% ethanol and incubated for three weeks in 8mM gadolinium contrast agent. Brains were then transferred to a 2ml Eppendorf tube filled with Fomblin and scanned in pairs using a three-dimensional gradient echo pulse sequence and an Agilent 7T DirectDrive MRI scanner, with acquisition parameters as follows; matrix 512x192x192 (reconstructed to 512x256x256); field of view 40x10x10 mm; repetition time/echo time (TR/TE) 30/10 ms; 20 signal averages; total scan time 8.2 hours. A 26mm radiofrequency coil was used for signal transmission and reception. Magnetic resonance images were processed blind to genotype using a combination of FSL^2^, ANTs^3^ and in-house C++ software utilizing the ITK library, available from https://github.com/spinicist/QUIT^4, 5^. In brief, multi-head scans were bias-field corrected^6^ before being split into individual sample images. Registration was then performed between each subject and the Dorr atlas image^7^ to ensure all samples were aligned. An average study template image was then constructed using MR images from all animals^8^. The resulting template was then non-linearly registered to the atlas image. All subject images were then non-linearly registered to the study template. The inverse transforms from the atlas to the study template and from the study template to each subject were applied to calculate the total brain volume and individual brain region of interest (ROI) volumes of each subject. ROIs match those found in the Dorr atlas^7^.

### **Histology**

Five 9 week old male littermate genotype trios were used for histological analysis except where indicated below. Perfused brains were removed from 70% ethanol and paraffin wax-embedded, then sections were cut from three different zones of the brain; Bregma ≈2.46 (prefrontal cortex); Bregma ≈ 0.75 (lateral ventricles and corpus callosum); Bregma ≈ -1.94 (hippocampus). Brains were processed by the University of Edinburgh Shared University Research Facilities (SURF), using a Leica RM2235 base sledge microtome. Twenty coronal sections of 10μm were cut for each block. Sections were mounted on to Superfrost Plus slides (ThermoFisher Scientific) and oven-dried. Two successive sections were used per location for each procedure.

To visualize cytoarchitecture by Nissl staining, sections were dewaxed in xylene, then rehydrated through graded alcohols. Rehydrated slides were incubated for 2 minutes in 0.2 % Cresyl fast violet solution containing 10 drops of acetic acid per 100 ml. Sections were dehydrated through graded alcohols, then cleared in xylene and cover-slipped with the xylene-based mounting solution DPX (Fisher Scientific).

To examine Parvalbumin-expressing neurons, sections were dewaxed and endogenous peroxidase activity quenched by incubating in methanol containing 1% hydrogen peroxide for 30 minutes. Sections were incubated in 10 mM sodium citrate buffer at room temperature, then microwaved for 20 minutes at high power. Sections were cooled on ice for 20 minutes, then blocked using 20% normal goat serum (Vector) for 1 hour. Sections were next incubated overnight at 4°C with mouse anti-Parvalbumin primary antibody (Sigma-Aldrich P3088, 1:400 dilution), followed by incubation at room temperature for one hour with biotinylated goat anti-mouse secondary antibody (Sigma-Aldrich, 1:200 dilution). Next, sections were incubated in Vectastain Elite Avidin-Biotin Complex (Vector Laboratories) for 30 minutes before visualization by incubation with 0.05% 3,3’-diaminobenzidine tetrahydrochloride (DAB, Sigma-Aldrich) containing 0.001% hydrogen peroxide. Finally, sections were dehydrated through graded alcohol, cleared in xylene, and cover slipped with DPX. To analyse the distribution of interneurons, the prefrontal cortex sections were initially separated into different regions of interest, distinguished using anatomical features and the Mouse Brain Atlas in stereotaxic coordinates^9^, then combined. In the hippocampal sections interneurons were counted in CA1 and the dentate gyrus, as well as the whole hippocampal area. ROIs were set at the same position on each section and the cells were counted manually, blind to genotype, using the Fiji ‘Cell Counter’ plugin.

To examine apoptotic cells, the same procedure was used except the primary antibody was specific for active (cleaved) caspase-3 (Sigma-Aldrich, AB3623, 1:70 dilution), the secondary antibody was biotinylated goat anti-rabbit (Sigma-Aldrich, 1:200 dilution), with nickel was added to the DAB solution. Sections were counterstained with Nuclear Fast Red (Vector Laboratories).

For cortical layer measurements in barrel cortex, brains were taken from three to five month old mice and immediately placed in 4% paraformaldehyde overnight. Fixed brains were sectioned using a cryostat (Leica) coronally at 50µm from rostral to caudal. Sections were mounted onto Superfrost slides (VWR) in gelatin solution. The slices were washed in acetone and water, then stained in 1% thionin-Nissl and dehydrated in increasing concentrations of alcohol, cleared in xylene and then coverslipped using DPX (Fluka).

**Image analysis**

For most purposes, images of brain sections were captured using a dotSlide scanner (Olympus). Equivalent areas of both hemispheres were quantified on each slide, blind to genotype, then averaged per animal. Cell density was determined within the regions of interest shown (Supplementary Figure 2), which were manually drawn and set using the Fiji region of interest manager, with area determined and cells counted using the Fiji ‘Cell Counter’ plugin.

For cortical layer measurements in barrel cortex, brain sections were imaged using a light microscope (Olympus). Barrel cortex could be identified by the presence of barrels in layer IV cortex (Bregma anterior-posterior 0.38 to -1.94mm) whilst sections of brain containing prefrontal cortex (Bregma 2.80 to 2.10mm) were identified using a mouse brain atlas^9^. Analysis of cortical thickness was performed within distinct brain areas. For cortical layer thickness, layers I, II/III, IV and V/IV were measured as distances perpendicular to the pial surface in addition to the total cortical thickness in barrel cortex. Total cortical thickness between the central sulcus and white matter for limbic cortex was measured for prefrontal cortex using Camera Lucida (Olympus).

**RNA sequencing**

Hippocampi, and cortices minus hippocampus, cerebellum and olfactory bulbs, were dissected from the right brain hemisphere mice at nine weeks of age. Samples were snap frozen in liquid nitrogen and stored at -80^o^C, then processed in batches of mixed genotypes to extract the RNA. Total RNA samples were assessed with a Fragment Analyser (Agilent) for quality and integrity of total RNA. Libraries were prepared using 100ng of each total RNA sample using the TruSeq Stranded mRNA Library Prep Kit (Illumina). Single end RNA Sequencing was carried out to a depth of approximately 60 to more than 100 million reads. Demultiplexing of sequencing reads was carried out using CASAVA (version 1.8.2, Illumina), with adapters trimmed using Skewer (version 0.1.116)^10^. Raw sequence reads were mapped to mouse reference genome mm10 using STAR (version 2.4.0h)^11^.

Raw counts at gene level were obtained using htseq-count^12^ (version 0.7.2, in the default union mode) on the alignment bam files and the Ensembl release 85 mouse gtf file. Differential gene expression was analysed using DESeq2 from the R statistical package^13^. Differential exon expression was analysed using DEXSeq^14^ (version 1.19.4) using exon counts obtained by running the script “dexseq_count.py” provided by the Deseq package. Adjusted-p-values were calculated via a Benjamini-Hochberg Procedure to get False Discovery Rate (FDR), the default in Deseq2 package. Raw count data for all samples were together subjected to a regularised logarithm transformation^10^ using the DESeq2 package version 1.16.1. For each heat map, the transformed counts for each gene were normalised to Z-scores across all samples. Heat maps of gene expression were generated using R (version 3.4.2) and RStudio (version 1.0.143).

**Expression-weighted cell-type enrichment (EWCE) analysis**

This analysis used the Karolinska Institute ‘Superset’ of RNASeq profiles generated from six independent single cell RNASeq studies of several brain regions and cell types. The Superset consists of 24 cellular classes generated by hierarchical clustering of nearly 9,970 mouse brain single cell RNASeq profiles (all generated by exactly the same method) followed by cell type identity assignment^15^. Profiles consist of a set of specificity values which provide a measure of gene expression enrichment (calculated from mean expression of each gene in a cellular class divided by its mean expression in all cellular classes) for each gene detected in that class^15^. Some cells were isolated from mouse cortex, hippocampus, striatum, hypothalamus and midbrain, while others were the result of specifically isolating cortical Parvalbumin-positive interneurons or oligodendrocytes from multiple brain regions including somatosensory cortex and hippocampus. The ages of the mice used to generate the profiles include embryonic and a range from P14 to P90. Each class and profile is therefore an amalgamation of single cell profiles from closely related cell types, not all necessarily from the same brain region or age. Superset profiles were downloaded from <http://hjerling-leffler-lab.org/data/scz_singlecell> using specificity table: ctd[[1]]$specificity and expression table: ctd[[1]]$mean_exp. Analysis was carried out in the R package using script downloaded from <https://github.com/NathanSkene/EWCE/> (version 0.99.2), and default options with Bonferroni multiple testing correction. The full list of expressed *Der1* cortex or hippocampus genes was used as background, as appropriate. The script was run with 10,000 repetitions. The Superset samples were sequenced at a lower depth than the *Der1* samples, and using unique molecular identifiers. Consequently only the most abundantly expressed genes (up to 14,581) were detected. For cortex, 1,794 of 2,125 dysregulated genes are present in the Superset profiles. For hippocampus, 151 of 175 dysregulated genes are present in the Superset profiles. *Der1* whole gene DESeq2 RNASeq data were used for this analysis.

**RNASeq deconvolution**

RNASeq deconvolution was carried out using the cell Karolinska Institute ‘Superset’ of RNASeq profiles^15^ described above under EWCE analysis as reference. Specificity value thresholds of 0.75 and 0.6 were set to ensure that the most highly enriched genes were used in this analysis, thus profile signatures consisted of genes with at least one specificity value in one cell type above these thresholds. To provide context, specificity value=1 represents 100% specificity for one cell class, the astrocyte marker Gfap exhibits a specificity value of 0.87 in the astrocyte/ependymocyte class, the oligodendrocyte marker Mbp exhibits a specificity value of 0.6 in the oligodendrocyte class, the interneuron marker Parvalbumin exhibits specificity values of 0.41 and 0.27 in the interneuron and striatal interneuron classes, respectively, and the synapse marker Dlg4 (Psd95) exhibits specificity values of ~0.1 in pyramidal neurons and <0.1 in other neuron classes^15^. This resulted in the use of 346 genes for threshold=0.75, and 752 genes for threshold=0.6, of which 285 and 653 are present in our wild-type RNASeq data.

Deconvolution was carried out using CIBERSORT Jar Version 1.06 (May 5^th^ 2017)^16^, available at the web interface <https://cibersort.stanford.edu>. The ability of CIBERSORT to accurately deconvolute the 24 cell types in the Superset was examined by creating artificial cell mixes by combining Superset gene expression values in various proportions from 0 to 0.5. CIBERSORT input was then compared to output to determine the efficiency of artificial sample deconvolution for the 24 cell types.

**Pathway analysis**

DESeq2 data were examined separately or combined with DEXSeq data by Ingenuity Pathway Analysis (IPA, Qiagen), using corrected p values and log2 fold changes, and the corresponding full list of expressed genes for each brain region as the background gene set. Human t(1;11) translocation neuron RNASeq data^1^ were similarly analysed using IPA. Pathway analysis of putative schizophrenia or depression risk genes was carried out using IPA and the full cortical gene expression list as the background gene set. Pathway analysis of dysregulated orthologues of putative schizophrenia or depression risk genes used the corresponding full list of expressed genes for each brain region as the background set. Adjusted p<0.05, and z>2 or z<-2 were used as thresholds throughout. Pathway analysis of dysregulated genes from cell class profiles used the full Superset profile as background. A specificity value threshold of 0.2 was set to ensure that a sufficient number of the most specific genes were used in the analysis. For context regarding specificity values see deconvolution, above. Where DEXSeq identified dysregulated sequences that did not unambiguously map to a single gene, or mass spectrometry identified peptides that could not be unambiguously mapped to a single protein (due to close homology with other proteins), all possible genes and proteins were included in the pathway analysis.

**Synaptosome preparation and mass spectrometry**

Synaptosomes were prepared from 8-10 week old *Der1* cortex and hippocampus (six wild-type, five heterozygous, five homozygous) as previously described^17^. Tissue was homogenized in HEPES buffer (5 mM HEPES, pH 7.4, 0.32 M sucrose supplemented with protease inhibitor cocktail, Roche) and centrifuged at 1000 x g for 10 min at 4°C. The supernatant was subsequently centrifuged in a 0.85/1.2 M sucrose gradient at 100,000 x g for 2 hours. Synaptosomes were recovered from the 0.85/1.2 M sucrose interface and concentrated by centrifugation at 18.000 x g for 30 min.

Samples were digested using filter aided sample preparation (FASP) with some modifications^18^. Briefly, 20 μg of each protein sample were incubated with 75 μL 2% SDS, 1 mM Tris(2-carboxyethyl)phosphine at 55°C for 1 hour, after which samples were incubated with 0.5 μL 200 mM methyl methanethiosulfonate for 15 min. Next, 200 μL 8 M Urea in Tris pH 8.8 were added and the samples were transferred to Microcon-30 filter tubes (Millipore). Samples were washed 4 times with 8M Urea in Tris buffer and 4 times with 50 mM ammonium bicarbonate by centrifugation at 14,000 x g for 10 min each. Proteins were digested with 0.7 μg Trypsin/Lys-C Mix (MS grade, Promega) overnight at 37°C. Peptides were eluted with 200 μL 50 mM ammonium bicarbonate, dried in SpeedVac and stored at -20°C.

Peptides were analysed by micro LC MS/MS using an Ultimate 3000 LC system (Dionex, Thermo Scientific) and the TripleTOF 5600 mass spectrometer (Sciex). Peptides were trapped on a 5 mm Pepmap 100 C18 column (300μm i.d., 5μmparticle size, Dionex) and fractionated on a 200 mm AlltimaC18 column (300μm i.d., 3μm particle size). The concentration of acetonitrile in the mobile phase was increased at a flow rate of 5μL/min from 5 to 18% in 88 min, to 25% at 98 min, 40% at 108 min and to 90% in 2 min. Peptides were electro-sprayed into the mass spectrometer with a micro-spray needle (at 5500 V). The mass spectrometer was operated in a data-independent mode, as described in^19^. Each cycle consisted of a parent ion scan of 150 msec and 8 Da MS/MS windows (80 msec scan time each), throughout a 450-770 m/z mass range. The collision energy for each window was calculated for a 2+ ion centered upon the window (spread of 15 eV).

The data were analysed with Spectronaut Pulsar v 12.0.20491.21.28109^20^ and using a spectral library created by data-dependent acquisition from hippocampal synapse-enriched samples containing spike-in iRT peptides (Biognosys). Cross-run normalization was enabled using local normalization strategy. Only peptides quantified with a Q-value ≤ 10^-2^ and 10^-3^ (for hippocampus and cortex datasets, respectively) across all samples in at least two groups were considered. Limma R package was used to Loess normalize protein abundance (‘normalizeCyclicLoess’ function, ‘fast’ method and 10 iterations). Volcano plots were generated using R (version 3.6.2). Protein were annotated to synaptic genes and sunburst plots were generated using SynGO 1.0 database and online tool^21^.

**Hippocampal Cell Culture and Electrophysiological recordings**

Primary hippocampal cultures were prepared from individual E17.5 DER littermate pups as described^22^. Briefly, hippocampi were dissected from pups, incubated in Papain, dissociated and grown in Neurobasal A growth medium containing 1% Rat Serum and supplemented with B-27, and maintained until Days In Vitro (DIV) 21.

Whole cell patch clamp recordings were performed as described^23^. Briefly, coverslips containing DIV 21 hippocampal neurons were transferred to a recording chamber with a constant (3-5ml/min) perfusion of external recording solution containing: 150 mM NaCl, 2.8 mM KCl, 10 mM HEPES, 2 mM CaCl_2_, 10 mM D-glucose and 100 µM glycine, pH 7.35, 320 mOsm. Tetrodotoxin citrate (300 nM) was included to block action-potential driven excitatory events. Patch-pipettes were pulled from borosilicate glass (Harvard Apparatus, Kent, UK) with a resistance of 3-5 MΩ, and filled with a K-gluconate-based internal solution containing: 141 mM Potassium Gluconate, 2.5 mM NaCl, 10 mM HEPES, 11 mM EGTA, pH 7.35). Currents were evoked by S-AMPA (50 µM) and NMDA (150 µM). All currents were recorded at room temperature, using an axopatch 200B amplifier (Molecular Devices, Union City, CA). Neurons were voltage-clamped at -60 mV. Whole-cell currents were analysed using WinEDR v3.2 software (John Dempster, University of Strathclyde, UK), with currents normalised to cell capacitance. For statistical analysis, n was taken as the number of pups, with n=3 WT, 4 HET and 3 HOM. A total of 12 WT, 14 HET and 11 HOM genotype coverslips were recorded from.

**Statistical analysis**

For analysis of MRI data, a multivariate general linear model 2-way MANCOVA statistical test was performed using SPSS statistics 22 (IBM) to determine group-level differences in brain ROI volumes with genotype as fixed effect, total brain volume and brain region as dependent variable, and littermate trio groupings as covariate.

For enrichment analysis, hypergeometric probabilities were calculated using keisan.casio.com/exec/system/1180573201. As with the pathway analysis, where DEXSeq identified dysregulated sequences that did not unambiguously map to a single gene, or mass spectrometry identified peptides that could not be unambiguously mapped to a single protein (due to close homology with other proteins), all possible genes and proteins were included in the enrichment analysis.

For the proteomic analysis, empirical Bayes moderated t-statistics with multiple testing correction by false discovery rate were performed on log-transformed protein abundances (‘eBayes’ and ‘topTable’ functions from Limma R package), as previously described^18, 19, 24, 25^. Proteins with a FDR adjusted p-value < 0.05 were considered significantly regulated for subsequent downstream analysis.

Other statistical analyses were carried out using GraphPad Prism, with statistical tests used stated in figure legends.

**Supplementary Table 1** (Excel file) Magnetic resonance imaging data. Both hemispheres, regional volumes (mm^3^) corrected to individual whole brain volumes, left and right hemispheres combined, separate hemispheres, regional volumes (mm^3^) corrected to individual whole brain volumes, left and right hemispheres considered separately

**Supplementary Table 2** (Excel file) RNA sequencing data. **a** DeSeq2 (whole gene differential expression) *Der1* cortex data, **b** DEXSeq (exon level differential expression) *Der1* cortex data, **c** DeSeq2 (whole gene differential expression) *Der1* hippocampus data, **d** DEXSeq (exon level differential expression) *Der1* hippocampus data. In each case data are provided with comparisons to human IPSC-derived cortical neuron cultures from members of the t(1;11) family^12^, two large-scale genome-wide association studies of schizophrenia^1, 2^, synapse genes from a large-scale schizophrenia CNV study^3^, two large-scale genome-wide association studies of depression^4, 6^, a large-scale genome-wide association study of bipolar disorder^5^, a large-scale genome-wide association study of Alzheimer's Disease^55^ where matches were found, and a large-scale genome-wide association study of cerebral cortex architecture^65^, where matches were found (references numbered according to main text). Overlaps are represented by a gene name in the relevant genetic study column. Non-overlaps are represented by empty cells, BaseMean, mean of normalised counts of all samples; p value, p value for wild-type versus heterozygous; adjusted p value, p value adjusted for multiple testing

**Supplementary Table 3** (Excel file) Dysregulated genes with conserved cAMP response elements according to <http://natural.salk.edu/creb/>.

**Supplementary Table 4** (Excel file) Ingenuity Pathway Analysis functions. **a** functions enriched for dysregulated genes in *Der1* cortex. All functions in the categories 'Molecular and cellular function' and 'Physiological system development and function' are included. Selected top relevant functions are provided in Table 1. Data are provided with comparisons to functions predicted from human IPSC-derived cortical neuron cultures from members of the t(1;11) family^12^ (reference numbered according to main text). Overlaps are represented by an x in the human neuron column. Non-overlaps are represented by empty cells, **b**, functions enriched for dysregulated genes in *Der1* hippocampus. All functions in the categories 'Molecular and cellular function' and 'Physiological system development and function' are included. Selected top relevant functions are provided in Supplementary Table 5. **c**, functions enriched for dysregulated genes in Superset cell classes. All functions in the categories 'Molecular and cellular function' and 'Physiological system development and function' are included. Selected top relevant functions are provided in Supplementary Table 6. **d**, functions enriched for dysregulated genes in human IPSC-derived cortical neuron cultures from members of the t(1;11) family. All functions in the categories 'Molecular and cellular function' and 'Physiological system development and function' are included. Selected top relevant functions are provided in Table 1. The genes listed for each function are dysregulated in the corresponding dataset.

**Supplementary Table 5** Top predicted relevant altered functions in heterozygous *Der1* mouse hippocampus.

| **Function (no. of molecules^a^)** | ***Der1* hippocampus score (no. of genes^b^)** |
| --- | --- |
| **General cell morphology** |  |
| Development of neurons (1,423) | p=2e-9 (33) |
| Morphology of neurons (1,123) | p=8e-7 (22) |
| Maturation of neurons (114) | p=8e-6 (7) |
| Abnormal morphology of neurons (923) | p=3e-5 (16) |
| Differentiation of neurons (648) | p=5e-4 (14) |
| **Cell contact** |  |
| Adhesion of neuronal cells (89) | p=8e-9 (9) |
| Formation of plasma membrane (406) | p=1e-8 (17) |
| Cell-cell contact (1,118) | p=1e-5 (22) |
| Cell-cell contact of neurons (24) | p=1e-5 (4) |
| Cell-cell adhesion of neurons (22) | p=3e-4 (3) |
| **Cytoskeleton** |  |
| Microtubule dynamics (2,247) | p=2e-4 (31) |
| Organization of cytoskeleton (2,624) | p=7e-4 (33) |
| **Cellular protrusions/neurites** |  |
| Neuritogenesis (1,067) | p=2e-6 (23) |
| Formation of cellular protrusions (1,645) | p=1e-4 (26) |
| Growth of neurites (910) | p=2e-4 (16) |
| Branching of cells (746) | p=2e-4 (14) |
| Extension of neurites (267) | p=5e-4 (8) |
| **Axons** |  |
| Extension of axons (134) | p=5e-3 (5) |
| Myelination of optic nerve (8) | p=2e-3 (2) |
| Myelination (8) | p=5e-3 (7) |
| **Dendrites** |  |
| Formation of dendrites (209) | p=2e-4 (8) |
| Dendritic growth/branching (446) | p=4e-4 (10) |
| Density of dendritic spines (143) | p=1e-3 (5) |
| Morphology of dendrites (138) | p=3e-3 (5) |
| Length of dendrites (47) | p=3e-3 (3) |
| **Cell proliferation** |  |
| Proliferation of epithelial cells (996) | p=3e-3 (14) |
| Neurogenesis of cerebral cortex (69) | p=5e-3 (3) |
| Proliferation of stem cells (372) | p=8e-3 (7) |
| **Transport** |  |
| Exocytosis (336) | p=9e-4 (8) |
| Transport of dopamine (76) | p=8e-4 (3) |
| Secretion of neurotransmitter (248) | p=1e-3 (7) |
| Release of neurotransmitter (510) | p=1e-3 (7) |
| Transport of 5-hydroxytryptamine (40) | p=2e-3 (2) |
| **Neurotransmission** |  |
| Developmental process of synapse (303) | p=3e-9 (16) |
| Neurotransmission (716) | p=8e-8 (20) |
| Synaptic transmission (558) | p=3e-7 (17) |
| Maturation of synapse (36) | p=3e-5 (4) |
| Miniature excitatory postsynaptic currents (71) | p=2e-4 (5) |
| Plasticity of synapse (170) | p=3e-4 (7) |
| Excitatory postsynaptic potential (166) | P=3e-4 (7) |
| Paired-pulse facilitation of synapse (55) | p=9e-4 (4) |
| Action potential of cells (238) | p=1e-3 (7) |
| Formation of excitatory synapses (14) | p=3e-3 (2) |

A full list of functions is provided in Supplementary Table 4b. Related functions are grouped, with top functions shown for each group. a, total number of molecules relating to each IPA function; b, number of dysregulated genes relating to each function

**Supplementary Table 6** Top predicted relevant altered cellular functions in cell classes from EWCE analysis.

| **Superset cell class** |  |
| --- | --- |
| **Function**  **(no. of molecules^a^)** | ***Der1* cortex score**  **(no. of genes^b^)** |
| ***Der1* cortex pyramidal CA1** |  |
| Long-term potentiation (539) | p=6e-6 (8) |
| Neurotransmission (773) | p=5e-5 (9) |
| Excitation of cerebral cortex cells (46) | p=6e-5 (3) |
| Synaptic transmission (601) | p=6e-5 (8) |
| Excitation of neurons (167) | p=9e-5 (4) |
| Remodelling of F-actin structure (7) | p=1e-4 (2) |
| Development of neurons (1,474) | p=2e-4 (12) |
| Neuritogenesis (1,110) | p=3e-4 (10) |
| AMPA mediated synaptic current (9) | p=3e-4 (2) |
| Activation of neurons (252) | p=3e-4 (4) |
|  |  |
| ***Der1* cortex pyramidal somatosensory** |  |
| Efflux of dopamine (63) | p=2e-5 (3) |
| Quantity of dense core vesicles | p=2e-4 (2) |
| Neurotransmission (773) | p=3e-4 (9) |
| Synaptic transmission 601) | p=3e-4 (8) |
| Exocytosis by eukaryotic cells (99) | p=1e-3 (3) |
| Fusion of synaptic vesicles (21) | p=1e-3 (2) |
| Action potential of neurons (198) | p=3e-3 (4) |
| Quantity of synapse (92) | p=3e-3 (3) |
| Accumulation of cortical actin filaments (1) | p=4e-3 (1) |
| Activation of parvocellular neurons (1) | p=4e-3 (1) |
|  |  |
| ***Der1* cortex interneurons** |  |
| Neurotransmission (773) | p=7e-6 (7) |
| Activation of neurons (252) | p=1e-5 (4) |
| Action potential of neurons (198) | p=5e-5 (4) |
| Excitation of neurons (167) | p=2e-4 (3) |
| GABA-mediated receptor currents (14) | p=2e-4 (2) |
| Fusion of plasma membrane (34) | p=4e-4 (2) |
| Excitation of cerebral cortex cells (46) | p=5e-4 (2) |
| Activation of enzyme (584) | p=9e-4 (5) |
| Accumulation of 2-arachidonoylglycerol (3) | p=1e-3 (1) |
| Activation of parvocellular neurons (1) | p=1e-3 (1) |
|  |  |
| ***Der1* cortex astrocytes/ependymocytes** |  |
| Fatty acid metabolism (1,492) | p=3e-8 (17) |
| Concentration of lipid (2,135) | p=3e-7 (19) |
| Uptake of amino acids (264) | p=8e-7 (6) |
| Uptake of glutamine family amino acid (133) | p=9e-7 (5) |
| Function of neuroglia (56) | p=2e-6 (5) |
| Mass of fat (24) | p=2e-6 (4) |
| Transport of amino acids (394) | p=3e-6 (7) |
| Concentration of fatty acid (721) | p=4e-6 (10) |
| Function of central nervous system (152) | p=4e-6 (6) |
| Function of oligodendrocytes (9) | p=5e-6 (3) |

A full list of functions is provided in Supplementary Table 5c, e. The most highly enriched genes that are dysregulated in *Der1* cortex for each cell class were used for IPA analysis, with specificity value cut-off=0.2 (SV=1 indicates 100% specificity, see methods for more context). In many cell classes the relatively low number of genes above this threshold was insufficient for meaningful pathway analysis. a, total number of molecules relating to each IPA function; b, number of dysregulated genes relating to each function

**Supplementary Table 7** (Excel file) Synaptosome mass spectrometry data. **a**, Mass spectrometry analysis of cortex synaptosomes isolated from wild-type (WT), heterozygous (HET) or homozygous (HOM) *Der1* mice, **b**, Mass spectrometry analysis of hippocampus synaptosomes isolated from wild-type (WT), heterozygous (HET) or homozygous (HOM) *Der1* mice, **c**, SynGo annotations. SD, standard deviation

**Supplementary table 8** Comparison between characteristics of the *Der1* mouse and pertinent characteristics of mutant mice that are known or proposed to be relevant to the t(1;11) translocation.

| **Mutant** | **Brain structure** | **Synapses & plasticity** | **Electrophysiology** | **Neuronal intracellular transport** |
| --- | --- | --- | --- | --- |
| ***Der1*** | ↑ hippocampal Parvalbumin-positive interneuron density  altered oligodendrocyte-myelin function^26^  no gross structural changes | ↑ surface/synaptic NMDA receptor expression in cultured hippocampal neurons^1^  altered Psd95 distribution indicative of an increased density of weaker synapses^1^  altered expression of genes involved in synapse formation, structure & function  altered expression of genes critical for synaptic plasticity and long-term potentiation, including the CREB signalling pathway | ↓ AMPA/NMDA ratio in cultured hippocampal neurons | ↑ NMDA receptor motility^1^  altered expression of genes required for vesicle transport and exo/endocytosis |
| **humanised DISC1-Boymaw & Boymaw-DISC1**^27^  endogenous mouse *Disc1* gene replaced with human *DISC1*-*Boymaw* or *Boymaw*-*DISC1* cDNA fusion transgenes (*Boymaw* is otherwise known as *DISC1FP1*) resulting in *Disc1* promoter-driven forced expression of putative chimeric proteins^28^ (whose expression in t(1;11) carriers remains to be established^1^) |  | ↓ cortical expression of NMDA receptor subunit GluN1 and Psd95^27^ |  |  |
| **Disc1△2-3**^29^  deletion of exons 2 & 3 from endogenous mouse *Disc1* gene, abolishes full-length Disc1 expression | ↓ density of Parvalbumin-positive interneurons in many cortical areas^30^, and in hippocampus^31^  no gross structural changes^29^ | catecholaminergic network dysfunction^32^  ↓ methamphetamine-induced dopamine release & ↑ dopamine receptor expression in nucleus accumbens^31^ | ↑ threshold for induction of long-term potentiation in hippocampus^29^ | ↓ dendritic ITPR1 mRNA transport in cultured hippocampal neurons^33^  ↓ synaptic vesicle exocytosis^34^ |
| **Disc1-LI**^35^  deletion of exons 1-3 from endogenous mouse *Disc1* gene, abolishes full-length Disc1 expression |  |  | altered parvalbumin-positive interneuron function^36^ |  |
| **Disc1_tr_**^37^  C-terminally truncated Disc1 (encoded by exons 1-8) fused to green fluorescent protein, expressed from transgenic mouse bacterial artificial chromosome under control of *Disc1* promoter | ↓ density of Parvalbumin-positive interneurons in hippocampus and medial prefrontal cortex, and displacement in dorsolateral prefrontal cortex^37^  ↑ lateral ventricle volume  ↓ cerebral cortex thickness  partial agenesis of corpus callosum^37^ | ↓ NMDA receptor GluN2A & GluN2B, ↑ GluN1 (trend) protein expression in hippocampus^38^ | ↑ long-term potentiation in Schaffer collateral commissural pathway  temporoammonic long-term potentiation abolished^39^  altered hippocampus-prefrontal cortex connectivity & reduced neurotransmitter release probability in the  glutamatergic hippocampal CA1–prefrontal cortex projection^38^ |  |
| **hDISC1**^40^  C-terminally truncated DISC1 (exons 1-8) transgene under inducible control of CaMKII promoter | ↓ density of Parvalbumin-positive cortical interneurons^41^  ↑ lateral ventricle volume^40^  altered oligodendrocyte specification^42, 43^ | ↓ cortical dopamine^41^ & dopamine D2 receptor binding in olfactory tubercle and nucleus accumbens (trend)^44^  ↑ dendritic spine density^41^  ↑ vesicular glutamate transporters in astrocytes^45^  ↑ NMDA receptor subunit GluN1, ↓ GluN2A in hippocampus^45^  altered homeostasis of dopamine and glutamate receptors in the nucleus accumbens^46^  reduced capacity of astrocytes to support dendritic and synaptic development^47^ | ↑ spontaneous excitatory postsynaptic currents in cultured cortical neurons^48^ | altered expression of proteins required for vesicular transport^49^ |
| **DN-DISC1**^50^  C-terminally truncated DISC1 (exons 1-8) transgene under control of CamKII promoter | ↓ density of Parvalbumin-positive cortical interneurons^50^  ↑ lateral ventricle volume^50^ |  | oscillations in hippocampal CA1^51^  abnormal action potentials, and dopaminergic regulation, in fast spiking parvalbumin-positive interneurons of prefrontal cortex^52^ |  |
| **DN-DISC1-PrP**^53^  C-terminally truncated DISC1 (exons 1-8) transgene under control of PrP promoter | no gross structural changes^53^ |  |  |  |
| **nes-DN-DISC1**^54^  C-terminally truncated DISC1 (exon 1-8) transgene inducibly expressed in neural precursor cells | ↑ density of Parvalbumin-positive interneurons in cingulate cortex, retrosplenial granular cortex, and motor cortex^54^ |  |  |  |
| **Disc1^Tm1Kara^** ^55^  natural deletion within mouse Disc1 exon 6 that introduces a premature termination codon, combined with targeted premature transcription termination signal in intron 8, abolishes full-length Disc1 expression and may express C-terminally truncated protein due to the termination codon within exon 7 | Parvalbumin-positive interneuron density unchanged^56^  ↓ prefrontal cortex volume^56^ | ↓ dendritic spine density & altered spine morphology in cultured hippocampal and cortical neurons^57^  altered hippocampal CREB signalling^58^ | ↓ short-term potentiation at hippocampal CA1-CA3 synapse  altered short-term plasticity at mossy fibre-CA3 circuit^58^  ↑ neuronal excitability in medial prefrontal cortex^59^  ↑ short-term depression & probable ↑ neurotransmitter release probability in medial prefrontal cortex^59^  ↑ spontaneous excitatory postsynaptic currents in cultured cortical neurons^48^  altered spontaneous inhibitory postsynaptic currents in cultured cortical neurons^48^ | ↓ synaptic vesicle volume at hippocampal CA3 synapses^58^  proteomic changes suggest effects upon synaptic vesicle transport^59^ |

The mutants fall into three main categories 1) recapitulation of the gene fusion between *DISC1* and *DISCFP1* (*Der1*, transgenic *Boymaw* fusions), 2) elimination of full-length *Disc1* expression (*Der1*, transgenic *Boymaw* fusions, Disc1△2-3, DISC1-LI, Disc1^Tm1Kara^), 3) transgenic overexpression of a truncated form of *Disc1* or *DISC1* encoded by exons 1-8 that was inferred to arise from the t(1;11) prior to discovery of the *DISC1*/*DISCFP1* gene fusion (Disc1_tr_, hDisc1, DN-DISC1, DN-DISC1-PrP, nes-DN-DISC1).


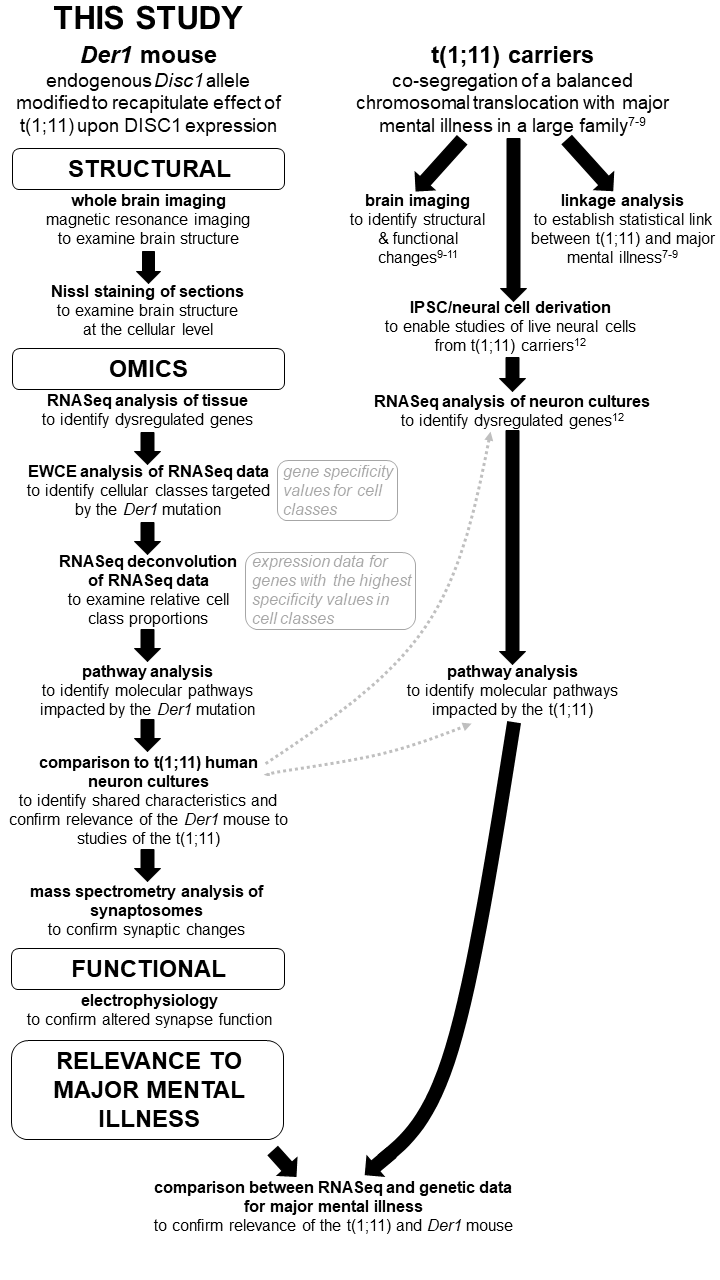


**Supplementary Fig. 1** Flowchart indicating the experimental approach taken. Superscript numbers indicate references according to the main (not supplementary) text.


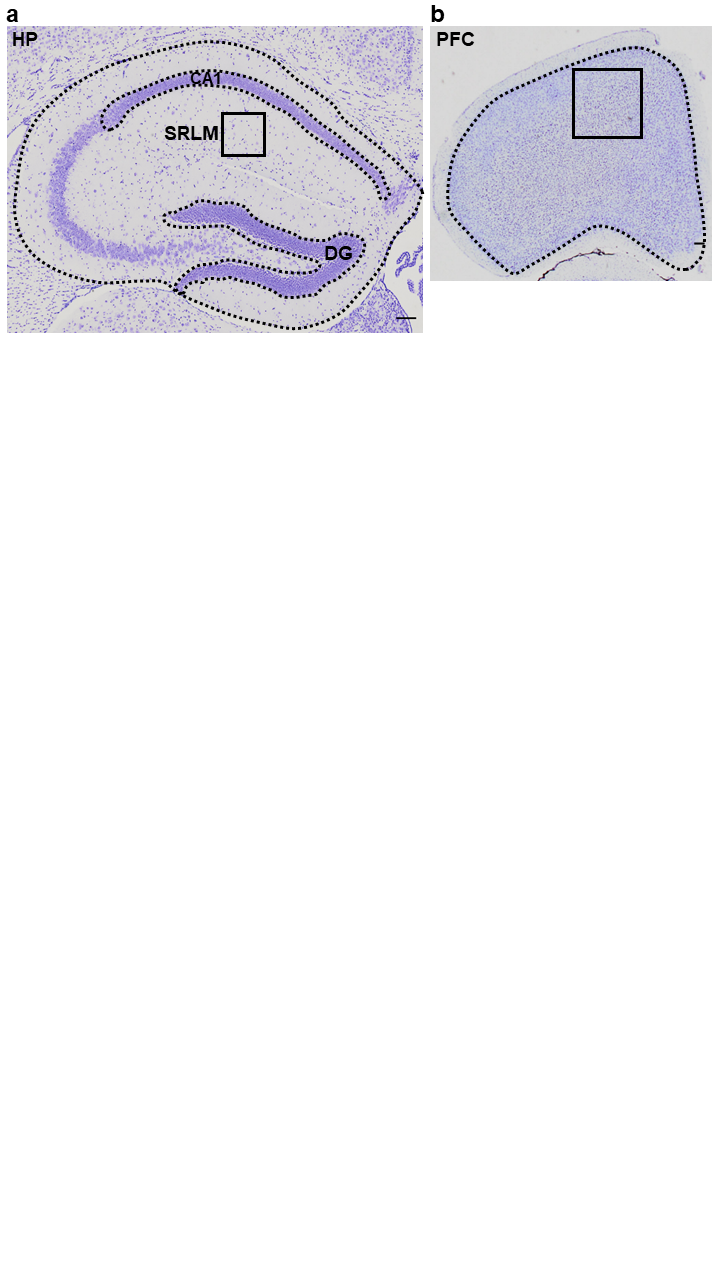


**Supplementary Fig. 2** Regions of interest for image analysis of cell density, Parvalbumin and cleaved Caspase 3. **a** Hippocampus (HP). The box indicates the region of the Stratum, Radiatum, Lacunosum and Moleculare (SRLM) in which cell density was quantified. Dotted lines outline the hippocampal formation, CA1 and the dentate gyrus (DG) used for quantification of Parvalbumin-positive cells and cells expressing cleaved Caspase 3. **b** Prefrontal cortex (PFC). The box indicates the region in which cell density was quantified. The dotted line outlines the region in which Parvalbumin-positive cells and cells expressing cleaved Caspase 3 were quantified. Scale bars, 100μm


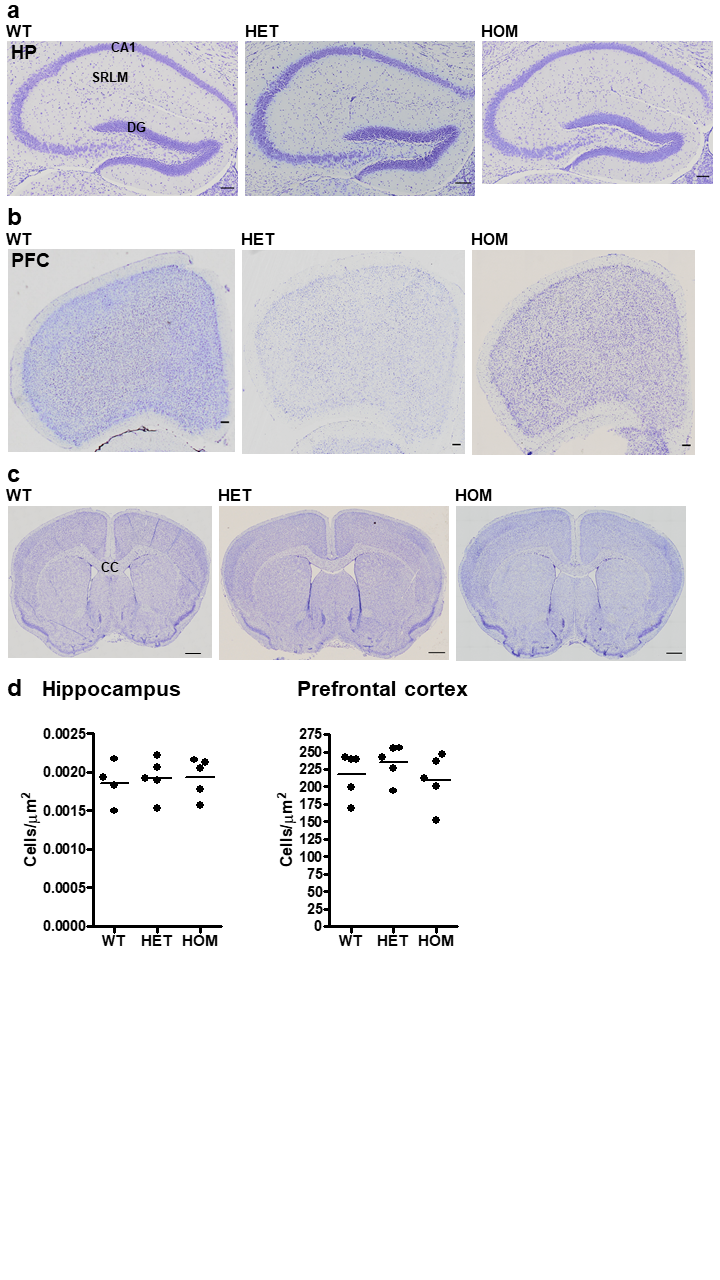


**Supplementary Fig. 3** Brain structure visualised by Nissl staining. Sections through hippocampus (HP) **a**, prefrontal cortex (PFC) **b**, and corpus callosum (CC) **c**, were stained with Nissl to visualise cell bodies and tissue structure. scale bars, 100μm in a and b, 500μm in c **d** Quantification of average cell density from both sides of the brain in hippocampal Stratum, Radiatum, Lacunosum and Moleculare, and PFC. Data were analysed by Kruskal-Wallis one-way ANOVA. Horizontal line on graphs, average of values for each sample; WT, wild-type; HET, heterozygous *Der1*; HOM, homozygous *Der1*


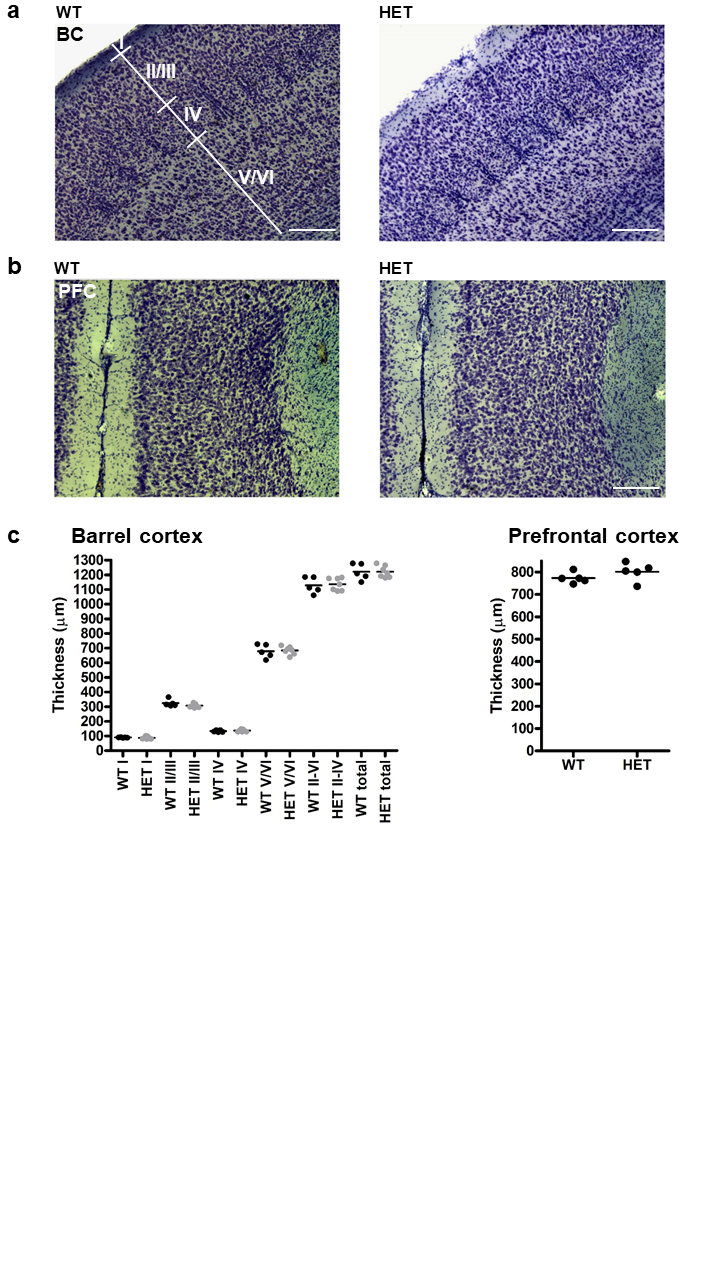


**Supplementary Fig. 4** Cortical layers visualised by Nissl staining. Barrel cortex was used to examine layering in detail because the individual cortical layers could not be distinguished in prefrontal cortex. Sections through barrel cortex (BC) **a**, and prefrontal cortex (PFC) **b**, were stained with Nissl to visualise cell bodies and tissue structure. Cortical layers and measurements taken are indicated. **c** Quantification of layer thickness in barrel cortex and PFC. Two-way ANOVA found no effect of genotype on layer thickness (F_1,40_=0.1959, p>0.05), nor any interaction between layer thickness and genotype (F_3,40_= 0.6631, p>0.05) in barrel cortex. Unpaired two-tailed t-test found no effect of genotype on cortical thickness in PFC (p=0.2). Horizontal line on graphs, average of values for each sample; scale bars, 200μm; WT, wild-type; HET, heterozygous *Der1*


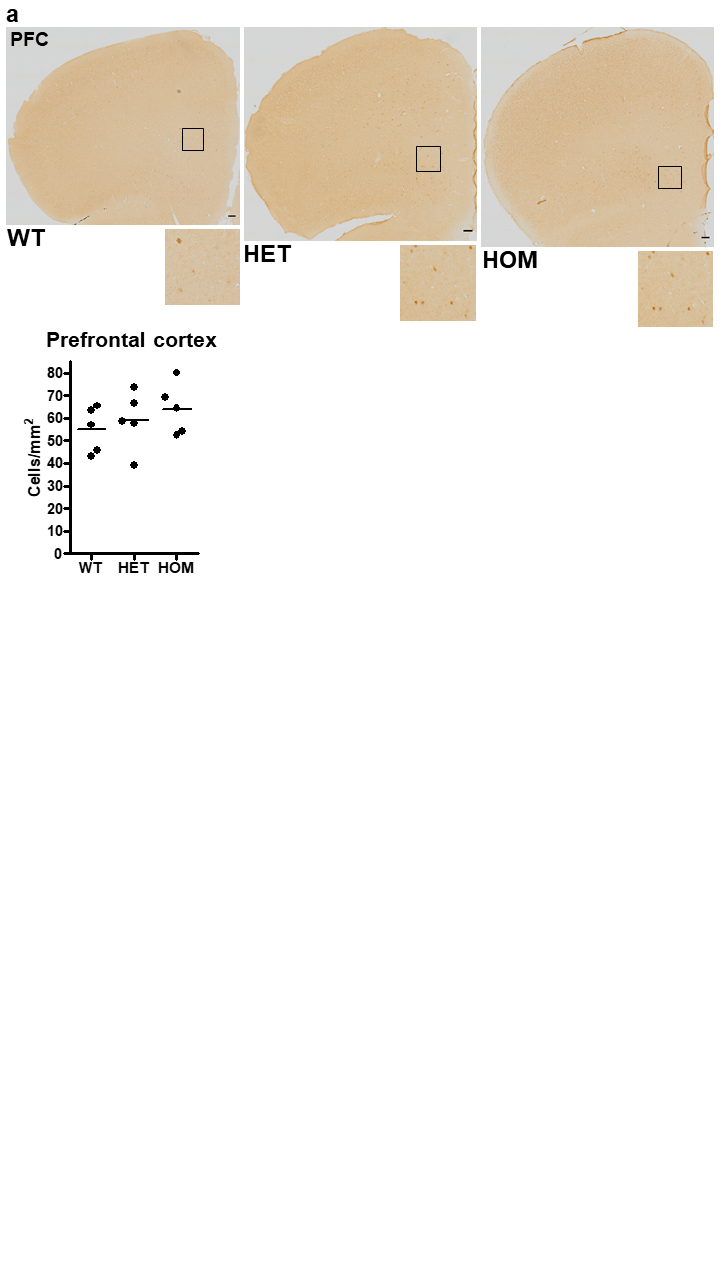


**Supplementary Fig. 5** No change in Parvalbumin-expressing interneuron density in *Der1* prefrontal cortex. **a** Prefrontal cortex (PFC) sections from nine week old mouse brain were stained with an antibody specific for Parvalbumin. Enlarged regions showing Parvalbumin-expressing interneurons are indicated by boxes. scale bars, 100μm **b** Average density of Parvalbumin-expressing interneurons from both sides of the brain. Data were analysed by Kruskal-Wallis one-way ANOVA. Horizontal line on graphs, average of values for each sample; WT, wild-type; HET, heterozygous *Der1*; HOM, homozygous *Der1*


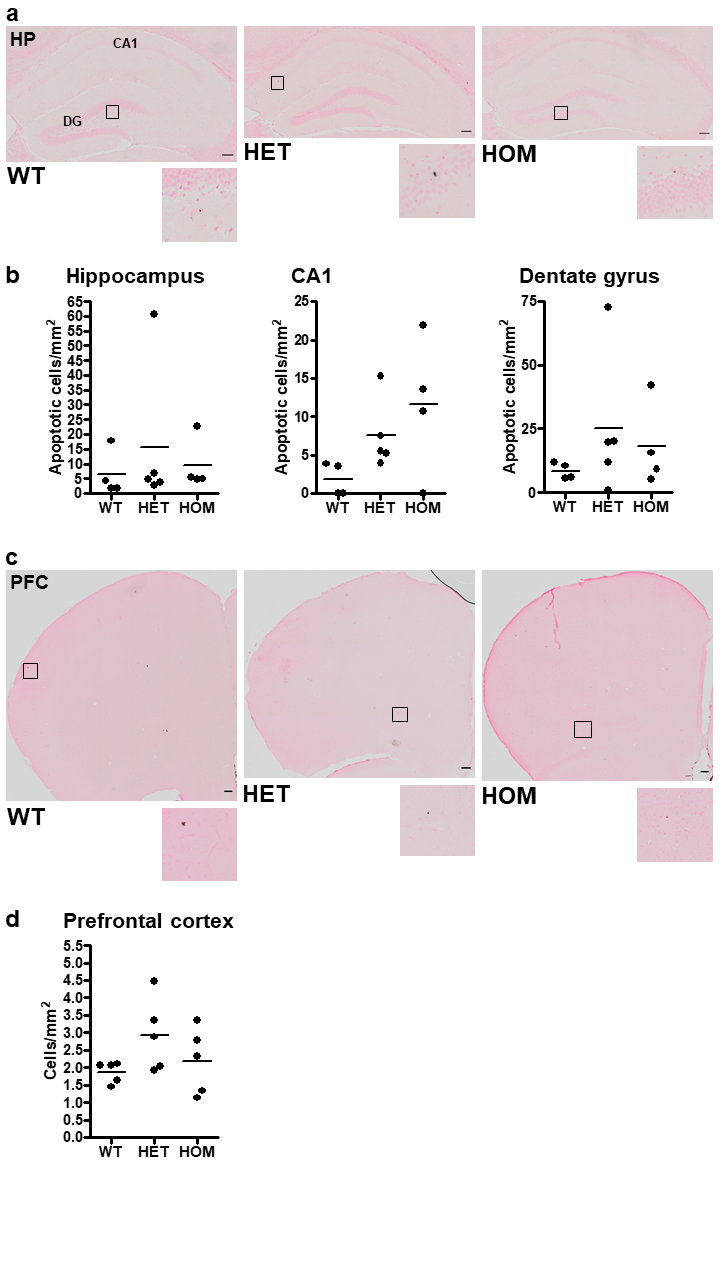


**Supplementary Fig. 6** Quantification of apoptotic cells. **a** Hippocampal (HP) sections from nine week old mouse brain were stained with an antibody specific for Activated Caspase 3 and counterstained with Nuclear Fast Red. Enlarged regions showing apoptotic cells are indicated by white boxes. **b** Average density of hippocampal apoptotic cells from both sides of the brain. Hippocampus refers to the whole hippocampal formation. **c** Prefrontal cortex (PFC) sections from nine week old mouse brain were stained with an antibody specific for Activated Caspase 3 and counterstained with Nuclear Fast Red. Enlarged regions showing apoptotic cells are indicated by boxes. **d** Average density of PFC apoptotic cells from both sides of the brain. Data were analysed by Kruskal-Wallis one-way ANOVA, p=0.06 for CA1. Horizontal line on graphs, average of values for each sample; WT, wild-type; HET, heterozygous *Der1*; HOM, homozygous *Der1*; DG, dentate gyrus; scale bars, 100μm
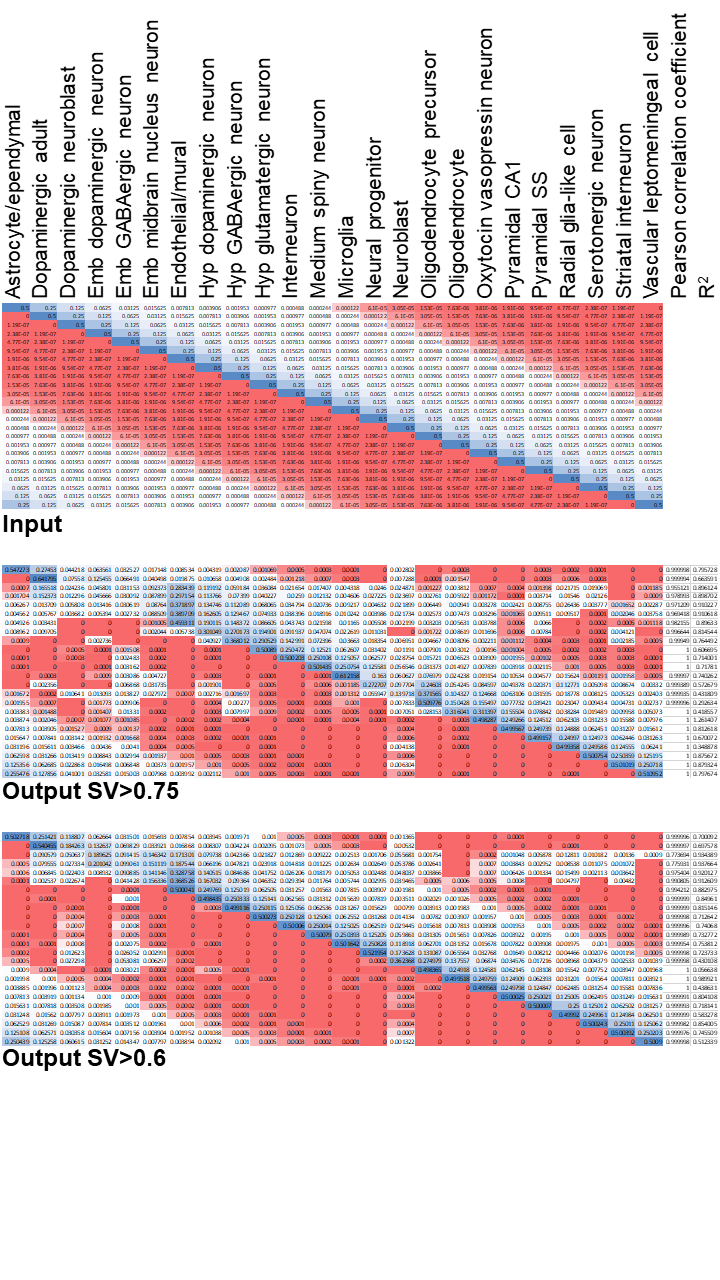


**Supplementary Fig. 7** Test deconvolution of the 24 Superset cell classes^15^. Reference profiles were generated using stringent specificity value (SV) thresholds of 0.75 or 0.6 to ensure that each cell class was represented by its most specific genes. *In silico* samples were created by mixing the thresholded gene expression profiles in proportions between 0 and 0.5. CIBERSORT input was compared to output and Pearson correlation coefficient and R^2^ calculated to assess the quality of deconvolution of each artificial sample.


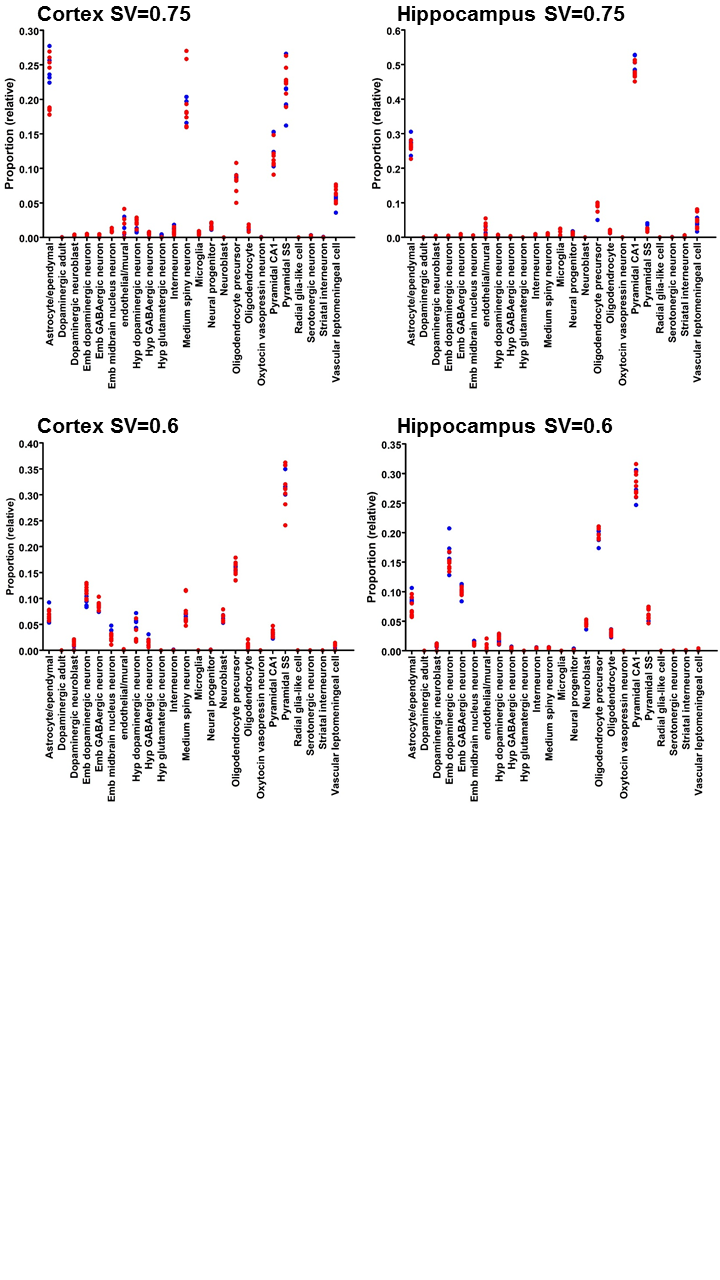


**Supplementary Fig. 8** Deconvolution of heterozygous *Der1* cortex and hippocampus RNASeq data using the Superset cell class profiles^15^ as reference. Reference profiles were generated using stringent specificity value (SV) thresholds of 0.75 or 0.6 to ensure that each cell class was represented by its most specific genes. Note that although the proportions change with the threshold set, and therefore the number of specific genes used for deconvolution, the relative proportions of each cell class do not differ between genotypes. Samples from embryonic cell types, neural progenitors and neuroblasts were not accurately deconvolved by CIBERSORT, thus their apparently high levels in the wild-type and *Der1* tissue are not an indication of true prevalence. Blue, wild-type; red, *Der1* heterozygote; Emb, embryonic


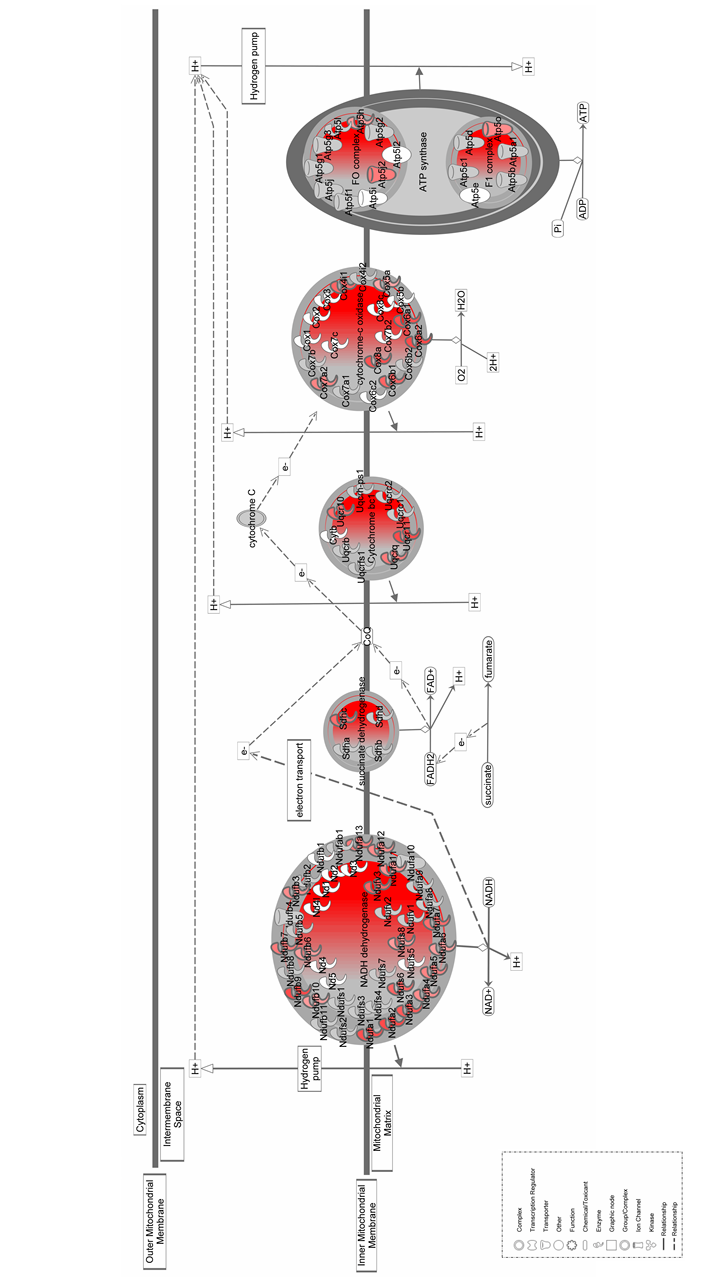


**Supplementary Fig. 9** *Der1* cortex gene dysregulation within the ‘Oxidative phosphorylation’ canonical pathway. Pathway impairment was predicted by IPA based on gene dysregulation at the whole gene level using DESeq2 data. Double outlines indicate protein complexes, the components of which can be found in Supplementary Table 2a, b. Colour intensity represents strength of gene expression change. green, downregulated; red, upregulated


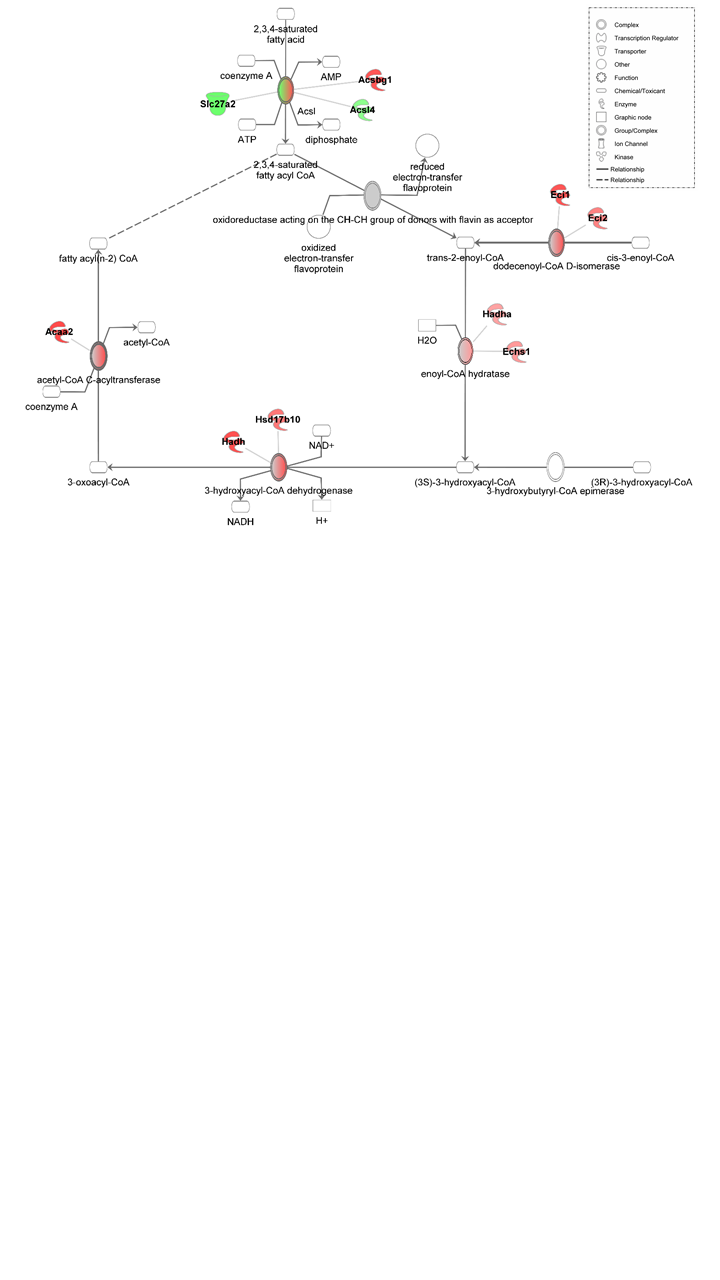


**Supplementary Fig. 10** *Der1* cortex gene dysregulation within the ‘Fatty acid β-oxidation I’ canonical pathway. Pathway impairment was predicted by IPA based on gene dysregulation at the whole gene level using DESeq2 data. Double outlines indicate enzyme complexes. To provide additional information, genes encoding relevant dysregulated enzymes and a transporter have been added to the pathway using the IPA ‘Build’ tool. Colour intensity represents strength of gene expression change, with graded colour within double outlined symbols representing overall direction of change within protein complexes. green, downregulated; red, upregulated


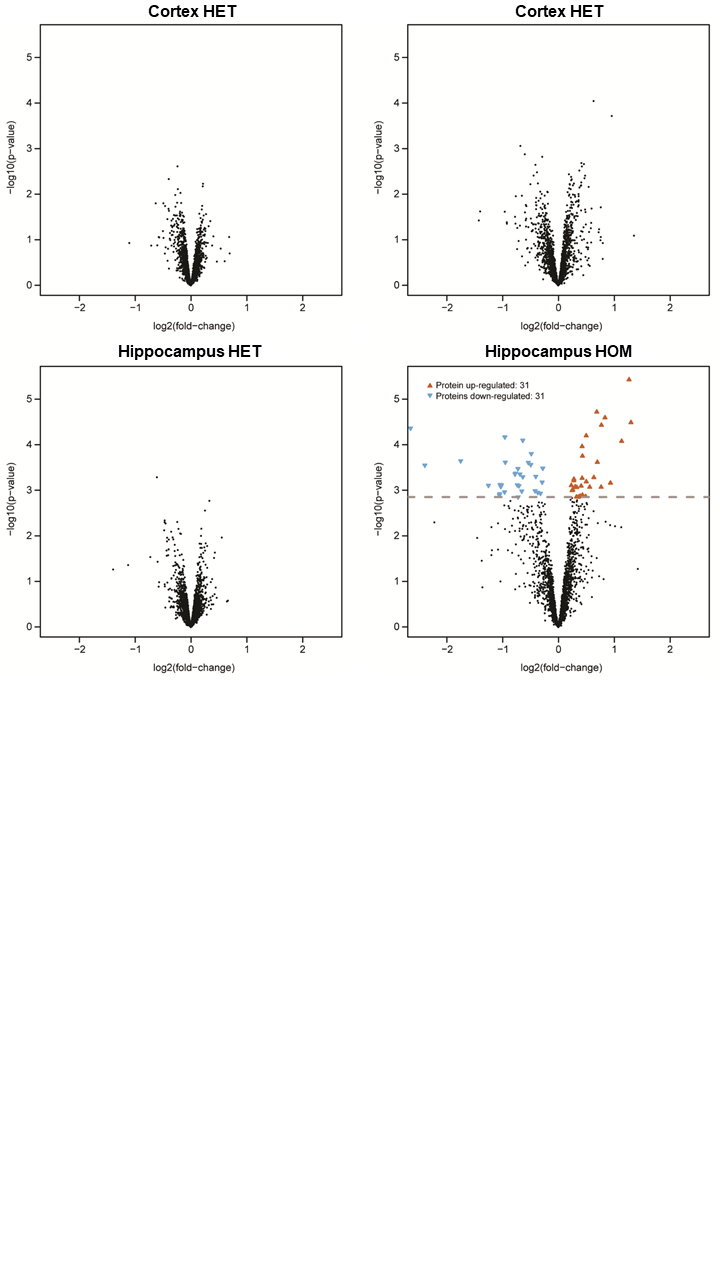


**Supplementary Fig. 11** Volcano plots showing differentially expressed synaptosomes proteins in comparisons between *Der1* mice and wild-type controls. No significant differences were found after multiple testing correction in cortex from heterozygous and homozygous *Der1* mice, nor in hippocampus from heterozygous *Der1* mice. In hippocampus from homozygous *Der1* mice, 62 proteins were found to be significantly dysregulated (FDR adjusted p-value ≤ 0.05) as indicated by the coloured dots above the dashed line.

**References**

1. Malavasi ELV, Economides KD, Grunewald E, Makedonopoulou P, Gautier P, Mackie S *et al.* DISC1 regulates N-methyl-D-aspartate receptor dynamics: abnormalities induced by a Disc1 mutation modelling a translocation linked to major mental illness. *Transl Psychiatry* 2018; **8**(1)**:** 184.

2. Jenkinson M, Beckmann CF, Behrens TE, Woolrich MW, Smith SM. Fsl. *NeuroImage* 2012; **62**(2)**:** 782-790.

3. Avants BB, Tustison NJ, Song G, Cook PA, Klein A, Gee JC. A reproducible evaluation of ANTs similarity metric performance in brain image registration. *NeuroImage* 2011; **54**(3)**:** 2033-2044.

4. Richetto J, Chesters R, Cattaneo A, Labouesse MA, Gutierrez AMC, Wood TC *et al.* Genome-Wide Transcriptional Profiling and Structural Magnetic Resonance Imaging in the Maternal Immune Activation Model of Neurodevelopmental Disorders. *Cereb Cortex* 2017; **27**(6)**:** 3397-3413.

5. Wood TC, Simmons C, Hurley SA, Vernon AC, Torres J, Dell'Acqua F *et al.* Whole-brain ex-vivo quantitative MRI of the cuprizone mouse model. *PeerJ* 2016; **4:** e2632.

6. Tustison NJ, Avants BB, Cook PA, Zheng Y, Egan A, Yushkevich PA *et al.* N4ITK: improved N3 bias correction. *IEEE Trans Med Imaging* 2010; **29**(6)**:** 1310-1320.

7. Dorr AE, Lerch JP, Spring S, Kabani N, Henkelman RM. High resolution three-dimensional brain atlas using an average magnetic resonance image of 40 adult C57Bl/6J mice. *NeuroImage* 2008; **42**(1)**:** 60-69.

8. Avants BB, Yushkevich P, Pluta J, Minkoff D, Korczykowski M, Detre J *et al.* The optimal template effect in hippocampus studies of diseased populations. *NeuroImage* 2010; **49**(3)**:** 2457-2466.

9. Paxinos G, Franklin KBJ. The mouse brain in stereotaxic co-ordinates. 4 edn. Elsevier Academic Press: Amsterdam, The Netherlands, 2013.

10. Jiang H, Lei R, Ding SW, Zhu S. Skewer: a fast and accurate adapter trimmer for next-generation sequencing paired-end reads. *BMC Bioinformatics* 2014; **15:** 182.

11. Dobin A, Davis CA, Schlesinger F, Drenkow J, Zaleski C, Jha S *et al.* STAR: ultrafast universal RNA-seq aligner. *Bioinformatics (Oxford, England)* 2013; **29**(1)**:** 15-21.

12. Anders S, Pyl PT, Huber W. HTSeq--a Python framework to work with high-throughput sequencing data. *Bioinformatics (Oxford, England)* 2015; **31**(2)**:** 166-169.

13. Love MI, Huber W, Anders S. Moderated estimation of fold change and dispersion for RNA-seq data with DESeq2. *Genome biology* 2014; **15**(12)**:** 550.

14. Anders S, Reyes A, Huber W. Detecting differential usage of exons from RNA-seq data. *Genome Res* 2012; **22**(10)**:** 2008-2017.

15. Skene NG, Bryois J, Bakken TE, Breen G, Crowley JJ, Gaspar HA *et al.* Genetic identification of brain cell types underlying schizophrenia. *Nature genetics* 2018; **50**(6)**:** 825-833.

16. Newman AM, Liu CL, Green MR, Gentles AJ, Feng W, Xu Y *et al.* Robust enumeration of cell subsets from tissue expression profiles. *Nat Methods* 2015; **12**(5)**:** 453-457.

17. Pandya NJ, Koopmans F, Slotman JA, Paliukhovich I, Houtsmuller AB, Smit AB *et al.* Correlation profiling of brain sub-cellular proteomes reveals co-assembly of synaptic proteins and subcellular distribution. *Sci Rep* 2017; **7**(1)**:** 12107.

18. Koopmans F, Pandya NJ, Franke SK, Phillippens I, Paliukhovich I, Li KW *et al.* Comparative Hippocampal Synaptic Proteomes of Rodents and Primates: Differences in Neuroplasticity-Related Proteins. *Front Mol Neurosci* 2018; **11:** 364.

19. He E, Lozano MAG, Stringer S, Watanabe K, Sakamoto K, den Oudsten F *et al.* MIR137 schizophrenia-associated locus controls synaptic function by regulating synaptogenesis, synapse maturation and synaptic transmission. *Human molecular genetics* 2018; **27**(11)**:** 1879-1891.

20. Bruderer R, Bernhardt OM, Gandhi T, Miladinovic SM, Cheng LY, Messner S *et al.* Extending the limits of quantitative proteome profiling with data-independent acquisition and application to acetaminophen-treated three-dimensional liver microtissues. *Mol Cell Proteomics* 2015; **14**(5)**:** 1400-1410.

21. Koopmans F, van Nierop P, Andres-Alonso M, Byrnes A, Cijsouw T, Coba MP *et al.* SynGO: An Evidence-Based, Expert-Curated Knowledge Base for the Synapse. *Neuron* 2019; **103**(2)**:** 217-234 e214.

22. Martel MA, Ryan TJ, Bell KF, Fowler JH, McMahon A, Al-Mubarak B *et al.* The subtype of GluN2 C-terminal domain determines the response to excitotoxic insults. *Neuron* 2012; **74**(3)**:** 543-556.

23. McKay S, Ryan TJ, McQueen J, Indersmitten T, Marwick KFM, Hasel P *et al.* The Developmental Shift of NMDA Receptor Composition Proceeds Independently of GluN2 Subunit-Specific GluN2 C-Terminal Sequences. *Cell Rep* 2018; **25**(4)**:** 841-851 e844.

24. Kammers K, Cole RN, Tiengwe C, Ruczinski I. Detecting Significant Changes in Protein Abundance. *EuPA Open Proteom* 2015; **7:** 11-19.

25. Smyth GK, Michaud J, Scott HS. Use of within-array replicate spots for assessing differential expression in microarray experiments. *Bioinformatics (Oxford, England)* 2005; **21**(9)**:** 2067-2075.

26. Vasistha NA, Johnstone M, Barton SK, Mayerl SE, Thangaraj Selvaraj B, Thomson PA *et al.* Familial t(1;11) translocation is associated with disruption of white matter structural integrity and oligodendrocyte-myelin dysfunction. *Molecular psychiatry* 2019.

27. Ji B, Higa KK, Kim M, Zhou L, Young JW, Geyer MA *et al.* Inhibition of protein translation by the DISC1-Boymaw fusion gene from a Scottish family with major psychiatric disorders. *Human molecular genetics* 2014; **23**(21)**:** 5683-5705.

28. Eykelenboom JE, Briggs GJ, Bradshaw NJ, Soares DC, Ogawa F, Christie S *et al.* A t(1;11) translocation linked to schizophrenia and affective disorders gives rise to aberrant chimeric DISC1 transcripts that encode structurally altered, deleterious mitochondrial proteins. *Human molecular genetics* 2012; **21**(15)**:** 3374-3386.

29. Kuroda K, Yamada S, Tanaka M, Iizuka M, Yano H, Mori D *et al.* Behavioral alterations associated with targeted disruption of exons 2 and 3 of the Disc1 gene in the mouse. *Human molecular genetics* 2011; **20**(23)**:** 4666-4683.

30. Umeda K, Iritani S, Fujishiro H, Sekiguchi H, Torii Y, Habuchi C *et al.* Immunohistochemical evaluation of the GABAergic neuronal system in the prefrontal cortex of a DISC1 knockout mouse model of schizophrenia. *Synapse* 2016; **70**(12)**:** 508-518.

31. Nakai T, Nagai T, Wang R, Yamada S, Kuroda K, Kaibuchi K *et al.* Alterations of GABAergic and dopaminergic systems in mutant mice with disruption of exons 2 and 3 of the Disc1 gene. *Neurochemistry international* 2014; **74:** 74-83.

32. Iritani S, Sekiguchi H, Habuchi C, Torii Y, Kuroda K, Kaibuchi K *et al.* Catecholaminergic neuronal network dysfunction in the frontal lobe of a genetic mouse model of schizophrenia. *Acta Neuropsychiatr* 2016; **28**(2)**:** 117-123.

33. Tsuboi D, Kuroda K, Tanaka M, Namba T, Iizuka Y, Taya S *et al.* Disrupted-in-schizophrenia 1 regulates transport of ITPR1 mRNA for synaptic plasticity. *Nature neuroscience* 2015; **18**(5)**:** 698-707.

34. Tang W, Thevathasan JV, Lin Q, Lim KB, Kuroda K, Kaibuchi K *et al.* Stimulation of Synaptic Vesicle Exocytosis by the Mental Disease Gene DISC1 is Mediated by N-Type Voltage-Gated Calcium Channels. *Front Synaptic Neurosci* 2016; **8:** 15.

35. Shahani N, Seshadri S, Jaaro-Peled H, Ishizuka K, Hirota-Tsuyada Y, Wang Q *et al.* DISC1 regulates trafficking and processing of APP and Abeta generation. *Molecular psychiatry* 2015; **20**(7)**:** 874-879.

36. Delevich K, Jaaro-Peled H, Penzo M, Sawa A, Li B. Parvalbumin Interneuron Dysfunction in a Thalamo-Prefrontal Cortical Circuit in Disc1 Locus Impairment Mice. *eNeuro* 2020; **7**(2).

37. Shen S, Lang B, Nakamoto C, Zhang F, Pu J, Kuan SL *et al.* Schizophrenia-related neural and behavioral phenotypes in transgenic mice expressing truncated Disc1. *J Neurosci* 2008; **28**(43)**:** 10893-10904.

38. Dawson N, Kurihara M, Thomson DM, Winchester CL, McVie A, Hedde JR *et al.* Altered functional brain network connectivity and glutamate system function in transgenic mice expressing truncated Disrupted-in-Schizophrenia 1. *Transl Psychiatry* 2015; **5:** e569.

39. Booth CA, Brown JT, Randall AD. Neurophysiological modification of CA1 pyramidal neurons in a transgenic mouse expressing a truncated form of disrupted-in-schizophrenia 1. *Eur J Neurosci* 2014; **39**(7)**:** 1074-1090.

40. Pletnikov MV, Ayhan Y, Nikolskaia O, Xu Y, Ovanesov MV, Huang H *et al.* Inducible expression of mutant human DISC1 in mice is associated with brain and behavioral abnormalities reminiscent of schizophrenia. *Molecular psychiatry* 2008; **13**(2)**:** 173-186, 115.

41. Ayhan Y, Abazyan B, Nomura J, Kim R, Ladenheim B, Krasnova IN *et al.* Differential effects of prenatal and postnatal expressions of mutant human DISC1 on neurobehavioral phenotypes in transgenic mice: evidence for neurodevelopmental origin of major psychiatric disorders. *Molecular psychiatry* 2011; **16**(3)**:** 293-306.

42. Katsel P, Tan W, Abazyan B, Davis KL, Ross C, Pletnikov MV *et al.* Expression of mutant human DISC1 in mice supports abnormalities in differentiation of oligodendrocytes. *Schizophr Res* 2011; **130**(1-3)**:** 238-249.

43. Katsel P, Fam P, Tan W, Khan S, Yang C, Jouroukhin Y *et al.* Overexpression of Truncated Human DISC1 Induces Appearance of Hindbrain Oligodendroglia in the Forebrain During Development. *Schizophrenia bulletin* 2018; **44**(3)**:** 515-524.

44. Pogorelov VM, Nomura J, Kim J, Kannan G, Ayhan Y, Yang C *et al.* Mutant DISC1 affects methamphetamine-induced sensitization and conditioned place preference: a comorbidity model. *Neuropharmacology* 2012; **62**(3)**:** 1242-1251.

45. Abazyan B, Nomura J, Kannan G, Ishizuka K, Tamashiro KL, Nucifora F *et al.* Prenatal interaction of mutant DISC1 and immune activation produces adult psychopathology. *Biological psychiatry* 2010; **68**(12)**:** 1172-1181.

46. Kim J, Horti AG, Mathews WB, Pogorelov V, Valentine H, Brasic JR *et al.* Quantitative Multi-modal Brain Autoradiography of Glutamatergic, Dopaminergic, Cannabinoid, and Nicotinic Receptors in Mutant Disrupted-In-Schizophrenia-1 (DISC1) Mice. *Mol Imaging Biol* 2015; **17**(3)**:** 355-363.

47. Xia M, Zhu S, Shevelkin A, Ross CA, Pletnikov M. DISC1, astrocytes and neuronal maturation: a possible mechanistic link with implications for mental disorders. *Journal of neurochemistry* 2016; **138**(4)**:** 518-524.

48. Holley SM, Wang EA, Cepeda C, Jentsch JD, Ross CA, Pletnikov MV *et al.* Frontal cortical synaptic communication is abnormal in Disc1 genetic mouse models of schizophrenia. *Schizophr Res* 2013; **146**(1-3)**:** 264-272.

49. Xia M, Broek JA, Jouroukhin Y, Schoenfelder J, Abazyan S, Jaaro-Peled H *et al.* Cell Type-Specific Effects of Mutant DISC1: A Proteomics Study. *Mol Neuropsychiatry* 2016; **2**(1)**:** 28-36.

50. Hikida T, Jaaro-Peled H, Seshadri S, Oishi K, Hookway C, Kong S *et al.* Dominant-negative DISC1 transgenic mice display schizophrenia-associated phenotypes detected by measures translatable to humans. *Proceedings of the National Academy of Sciences of the United States of America* 2007; **104**(36)**:** 14501-14506.

51. Altimus C, Harrold J, Jaaro-Peled H, Sawa A, Foster DJ. Disordered ripples are a common feature of genetically distinct mouse models relevant to schizophrenia. *Mol Neuropsychiatry* 2015; **1**(1)**:** 52-59.

52. Cardarelli RA, Martin R, Jaaro-Peled H, Sawa A, Powell EM, O'Donnell P. Dominant-Negative DISC1 Alters the Dopaminergic Modulation of Inhibitory Interneurons in the Mouse Prefrontal Cortex. *Mol Neuropsychiatry* 2018; **4**(1)**:** 20-29.

53. Niwa M, Jaaro-Peled H, Tankou S, Seshadri S, Hikida T, Matsumoto Y *et al.* Adolescent stress-induced epigenetic control of dopaminergic neurons via glucocorticoids. *Science (New York, NY* 2013; **339**(6117)**:** 335-339.

54. Deng D, Jian C, Lei L, Zhou Y, McSweeney C, Dong F *et al.* A prenatal interruption of DISC1 function in the brain exhibits a lasting impact on adult behaviors, brain metabolism, and interneuron development. *Oncotarget* 2017; **8**(49)**:** 84798-84817.

55. Koike H, Arguello PA, Kvajo M, Karayiorgou M, Gogos JA. Disc1 is mutated in the 129S6/SvEv strain and modulates working memory in mice. *Proceedings of the National Academy of Sciences of the United States of America* 2006; **103**(10)**:** 3693-3697.

56. Kvajo M, McKellar H, Arguello PA, Drew LJ, Moore H, MacDermott AB *et al.* A mutation in mouse Disc1 that models a schizophrenia risk allele leads to specific alterations in neuronal architecture and cognition. *Proceedings of the National Academy of Sciences of the United States of America* 2008; **105**(19)**:** 7076-7081.

57. Lepagnol-Bestel AM, Kvajo M, Karayiorgou M, Simonneau M, Gogos JA. A Disc1 mutation differentially affects neurites and spines in hippocampal and cortical neurons. *Molecular and cellular neurosciences* 2013; **54:** 84-92.

58. Kvajo M, McKellar H, Drew LJ, Lepagnol-Bestel AM, Xiao L, Levy RJ *et al.* Altered axonal targeting and short-term plasticity in the hippocampus of Disc1 mutant mice. *Proceedings of the National Academy of Sciences of the United States of America* 2011; **108**(49)**:** E1349-1358.

59. Crabtree GW, Sun Z, Kvajo M, Broek JA, Fenelon K, McKellar H *et al.* Alteration of Neuronal Excitability and Short-Term Synaptic Plasticity in the Prefrontal Cortex of a Mouse Model of Mental Illness. *J Neurosci* 2017; **37**(15)**:** 4158-4180.
